# Conserved function of the matriptase-prostasin proteolytic cascade during epithelial morphogenesis

**DOI:** 10.1371/journal.pgen.1007882

**Published:** 2019-01-02

**Authors:** Leonard Drees, Tatiana Königsmann, Martin H. J. Jaspers, Ralf Pflanz, Dietmar Riedel, Reinhard Schuh

**Affiliations:** 1 Research Group Molecular Organogenesis, Max-Planck-Institute for Biophysical Chemistry, Göttingen, Germany; 2 Research Group Mass Spectrometry, Max-Planck-Institute for Biophysical Chemistry, Göttingen, Germany; 3 Electron Microscopy Group, Max-Planck-Institute for Biophysical Chemistry, Göttingen, Germany; New York University, UNITED STATES

## Abstract

Extracellular matrix (ECM) assembly and remodelling is critical during development and organ morphogenesis. Dysregulation of ECM is implicated in many pathogenic conditions, including cancer. The type II transmembrane serine protease matriptase and the serine protease prostasin are key factors in a proteolytic cascade that regulates epithelial ECM differentiation during development in vertebrates. Here, we show by rescue experiments that the *Drosophila* proteases Notopleural (Np) and Tracheal-prostasin (Tpr) are functional homologues of matriptase and prostasin, respectively. Np mediates morphogenesis and remodelling of apical ECM during tracheal system development and is essential for maintenance of the transepithelial barrier function. Both Np and Tpr degrade the zona pellucida-domain (ZP-domain) protein Dumpy, a component of the transient tracheal apical ECM. Furthermore, we demonstrate that Tpr zymogen and the ZP domain of the ECM protein Piopio are cleaved by Np and matriptase *in vitro*. Our data indicate that the evolutionarily conserved ZP domain, present in many ECM proteins of vertebrates and invertebrates, is a novel target of the conserved matriptase-prostasin proteolytic cascade.

## Author summary

Epithelial tissue covers the outside of the animal body and lines internal organs. Its disorganization is the source of approximately 90% of all human cancers. Elaboration of the basic epithelial characteristics has led to an understanding of how complex structures such as the branched tubular networks of vertebrate lung or invertebrate tracheal system are organized. Aside from obvious morphological differences, specific compositions of the epithelial extracellular matrix (ECM) have been noted. For example, while the flexible ECM of the vertebrate skin mainly consists of collagen and elastic fibers, the rigid ECM of invertebrates is chitin-based to serve as an inflexible exoskeleton. We show that a central regulator of ECM differentiation and epithelial development in vertebrates, the matriptase-prostasin proteolytic cascade (MPPC), is conserved and essential for both *Drosophila* ECM morphogenesis and physiology. The functionally conserved components of the MPPC mediate cleavage of zona pellucida-domain (ZP-domain) proteins, which play crucial roles in organizing apical structures of the ECM in both vertebrates and invertebrates. Our data indicate that ZP-proteins are molecular targets of the conserved MPPC and that cleavage within the ZP-domains is a conserved mechanism of ECM development and differentiation.

## Introduction

Epithelial development establishes the basis for normal body shape and organ function. Sheets of epithelial cells separate different chemical milieus inside the body. They protect the body from the outside and organize into elaborate complex structures such as stratified epithelia and branched tubules [1,2]. Tissues mediate these diverse functions by controlling the paracellular flow of water-soluble molecules and by generating an extracellular matrix (ECM) that is critical both for organ shape and function as well as protecting organs from their surroundings [3,4]. In fact, epithelial tissue defects underlie approximately 90% of all human cancers [1].

Members of the type II transmembrane serine protease (TTSP) protein family play critical roles in epithelial development and cancer progression [5–7]. The mammalian TTSP matriptase is a key regulator of epithelial tissues (for reviews see [8,9]). Its dysregulation causes spontaneous squamous cell carcinomas and increased susceptibility to carcinogen-induced tumorigenesis [10]. Also, elevated matriptase expression is a key initiator and inducer in cartilage destruction in osteoarthritis [11]. Matriptase-deficient mice exhibit a variety of epidermal defects including a disruption of the stratum corneum ECM architecture and a compromised epithelial barrier function leading to fetal death by dehydration [12,13]. In the epidermis, matriptase activates the membrane-anchored serine protease prostasin by proteolytic cleavage, which is required to initiate a cascade in epithelial development (for review see [8]). In contrast to matriptase, which undergoes rapid auto-activation *in vitro* [14], prostasin zymogen is incapable of auto-proteolysis and requires matriptase for activation in most tissues [15]. The combined matriptase-prostasin proteolytic cascade is implicated in regulation of epithelial sodium channels [16], control of tight junction assembly and function [17], as well as ECM formation and degradation [8,18]. Thus, the matriptase-prostasin cascade appears to be fundamental for normal epithelial development, and its deregulation is linked to many pathogenic conditions.

The *Drosophila* tracheal system is an archetypal model for both epithelial development and ECM formation in invertebrates (for reviews see [3,19]). Its development is initiated by the differentiation of tracheal cell groups from ectodermal cells. These tracheal cells form tubes that branch out in a stereotyped pattern, and specific branches fuse to form a three-dimensional tubular network [20]. During embryogenesis, the tracheal lumen is filled with apical ECM (aECM), which includes a chitin matrix, chitin deacetylation proteins (Serpentine and Vermiform; [21]), as well as the zona pellucida (ZP)–domain proteins Piopio (Pio) and Dumpy (Dpy; [19]). These luminal matrix components are expressed in spatially and temporally restricted patterns and provide an essential, physical, cable-like scaffold to shape tube structure and organ design [22]. While this first wave of aECM is degraded near the end of embryogenesis, a second wave of matrix components organizes a unique, mature aECM, referred to as the taenidial folds. These structures are chitin-containing protrusions at the apical side of tracheal cells that coalesce into a helical design running perpendicular to the tube length along the lumen of tracheal branches [19]. The taenidial folds act as tube stiffeners to prevent collapse, while tolerating some expansion and contraction of the tubes at the same time. This scaffold becomes most significant once the luminal aECM components are degraded near the end of embryogenesis. The clearance of ECM material and the establishment of an osmotic pressure within the tubes are subsequently prerequisites for the gas filling of the tracheal tubes [23,24]. The osmotic pressure depends on an intact transepithelial barrier function mediated by septate junctions (SJs). These multi-protein complexes are localized in apico-lateral membranes of epithelial cells [25,26]. The gas filling, also known as liquid clearance (LC), of the tracheal tubes is the key event for respiratory organ function, allowing the transport of gases with the beginning of larval development.

Here we report that the TTSP Notopleural (Np) is the *Drosophila* functional homologue of human matriptase. We show that Np is essential for degradation of the Dpy cable in the tracheal lumen, gas filling of the tracheal system, and the proper formation of the taenidial folds. Furthermore, *Np* is critical for the maintenance of the transepithelial barrier function. In addition, we identified Tracheal-prostasin (Tpr) as a *Drosophila* functional homologue of human prostasin. Tpr acts in combination with Np in the developing tracheal system. We show that Np and its human functional homologue are capable of mediating Tpr zymogen activation. Both proteins act by cleaving the aECM protein Pio within the conserved ZP domain. We conclude that ZP-domain proteins of the ECM are targets of the Np- and matriptase-mediated proteolytic pathways in both invertebrates and vertebrates.

## Results

### *Notopleural* is essential for liquid clearance of the tracheal tubes

**Fig 1:**
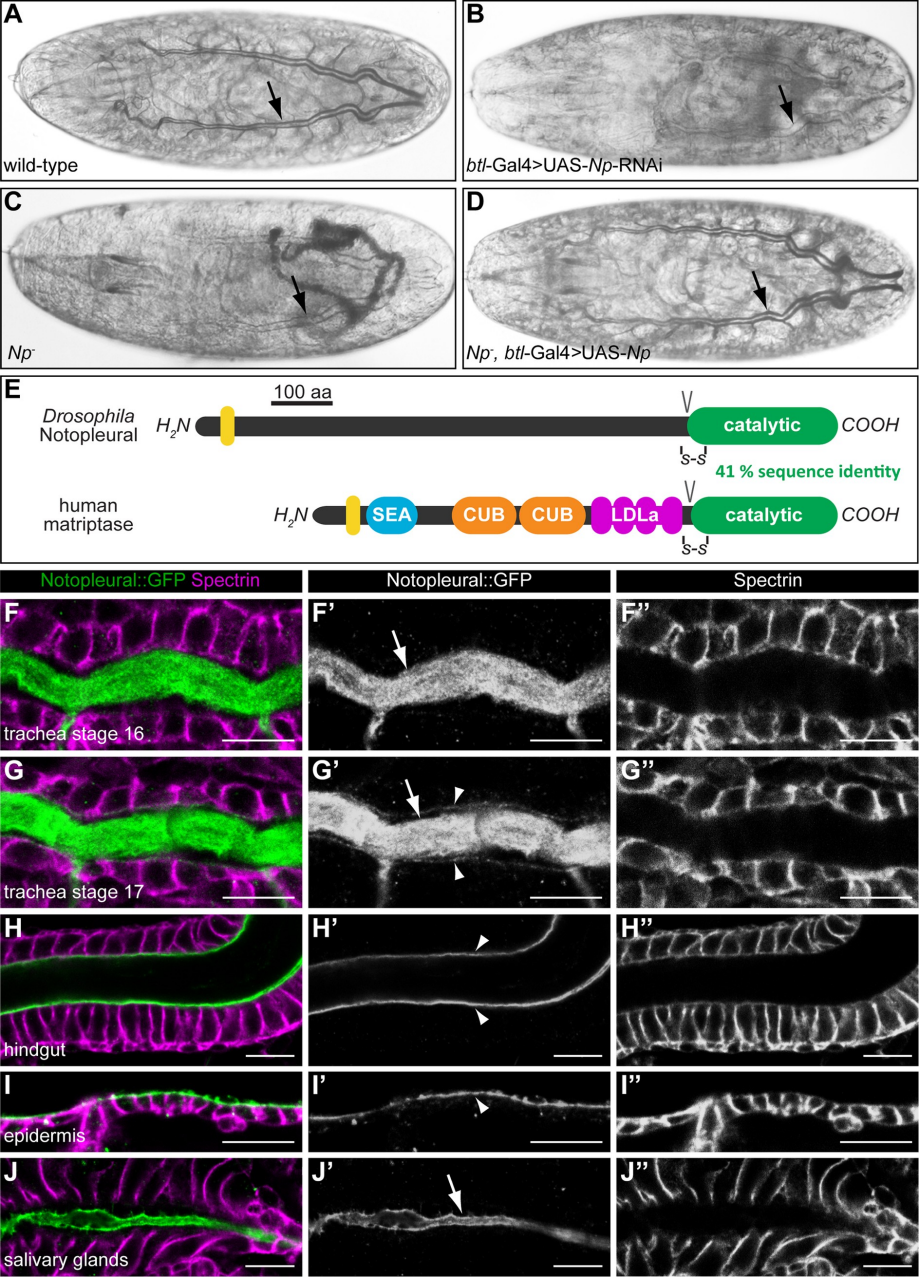
*Notopleural* is required for embryonic tracheal gas filling and encodes a serine protease related to human matriptase. (A-D) Bright field light microscopic images of stage 17 wild-type (A), *btl-*Gal4; UAS-RNAi-*GD13443* (B), *Np*^*P6*^*/Np*^*C2*^ mutant (C) and *Np*^*P6*^,*btl*-Gal4/*Np*^*C2*^,UAS-*Np* mutant (D) embryos. Wild-type embryos show gas filled tracheal tubes at the end of embryogenesis (arrow in A). RNAi-mediated tracheal knock-down of *CG34350* (*Np*) leads to lack of tracheal gas filling (arrow in B). *Np*^*P6*^*/Np*^*C2*^ mutant embryos lack gas filling (arrow in C) while *Np* mutant embryos with tracheal expression of *Np* show normal gas filling of the tracheal system (arrow in D). (E) Schema showing the protein domain organisations of *Drosophila* Np and human matriptase. The transmembrane domains (yellow), the SEA (sea urchin sperm protein/enteropeptidase/agrin), CUB (Cls/Clr, urchin embryonic growth factor, bone morphogenetic protein-1), LDLa (low-density lipoprotein receptor class A) and the catalytic protease domains are shown. Conserved disulphide bridges (-S-S-) and zymogen activation cleavage sites (V) are indicated. (F-J”) Confocal LSM images of whole-mount antibody stainings of *Np*::GFP embryos at stage 16 (F-F”, H-J”) and stage 17 (G-G”) stained with anti-Spectrin (magenta) and anti-GFP (green, Np::GFP) antibodies. Np::GFP is expressed in the tracheal system (F-G”), the hindgut (H-H”), the epidermis (I-I”) and the salivary glands (J-J”). Np::GFP is localized in the tracheal lumen during stage 16 (arrow in F’) and 17 (arrow in G’) and localizes to the apical membrane of tracheal cells during stage 17 (arrowheads in G’). In the hindgut and epidermis, Np::GFP is localized exclusively at the apical cell membranes (arrowheads in H’ and I’). In the salivary glands, Np::GFP is localized exclusively in the lumen (arrow in J’). Scale bars correspond to 10 μm.

We initially identified the gene *CG34350* in an RNA interference (RNAi) screen for genes required for gas filling of the tracheal tubes, a process referred to as liquid clearance (LC) [27]. In contrast to wild-type embryos, which undergo LC during stage 17 (Fig 1A), UAS/Gal4-mediated [28] pan-tracheal expression of an RNAi-transgene targeting *CG34350* (*btl*-Gal4; UAS-RNAi-*GD13443* [29]) leads to the complete lack of LC (Fig 1B). Such embryos hatch but die during the first instar larval stage. Mesodermal (*mef2*-Gal4) or endodermal (*Y48*-Gal4) RNAi-mediated *CG34350* knockdown, which serve as controls, lead to normal, fertile flies. Thus, the RNAi-mediated LC phenotype is specific, suggesting that *CG34350* is required for normal function of the tracheal system.

*CG34350* is identical with the gene *Notopleural* (*Np)*, which was identified *via* a dominant mutation [30]. We therefore refer to *CG34350* as *Np*. Using the CRISPR/Cas9 system we generated the *Np* loss-of-function alleles *Np*^*P6*^ and *Np*^*C2*^ (S1A–S1C Fig). Both new *Np* alleles fail to complement each other as well as Df(2R)BSC271, which deletes the chromosomal region 44F12-45A12 including the *Np* gene. Furthermore, *Np*^*P6*^ and *Np*^*C2*^ mutant embryos die during late stage 17 before hatching and lack LC of the tracheal tubes both in homozygous and hemizygous conditions (Fig 1C). Ectopic tracheal *Np* expression from a *Np* cDNA using the UAS/Gal4 expression system rescues the LC phenotype of *Np* mutant embryos, i.e. the tracheal system fills with gas (Fig 1D). These results indicate that *Np* has an essential function for normal gas filling of the tracheal system.

### *Notopleural* encodes a type II transmembrane serine protease related to human matriptase

In order to explore the molecular nature of Np, we examined its sequence. *Np* encodes a putative 1041 amino acid large protein. *In silico* analysis predicts a type II transmembrane serine protease (TTSP), which consists of a cytoplasmic amino-terminal domain, a transmembrane domain followed by an extracellular stem, and a carboxy-terminal trypsin-like serine protease catalytic domain (Fig 1E).

TTSPs are expressed as single-chain, catalytically inactive zymogens. Their catalytic domains are activated by proteolytic cleavage between the stem regions and protease domains. Following cleavage, the catalytic domains remain linked to the stems *via* a conserved disulfide bond, resulting in activated two-chain TTSPs, which remain membrane associated *via* their amino-terminal transmembrane domains [6,31]. When compared to human proteins, Np shows the highest degree of sequence similarity to the TTSP matriptase. They share a sequence identity of 41% within their catalytic domains (S2 Fig), while the extracellular stems and the cytoplasmic domains of both proteins show no sequence similarities (Fig 1E). These observations imply that *Np* encodes a putative TTSP with sequence homology to the human TTSP matriptase.

### Notopleural is a component of the apical extracellular matrix

To visualize *Np* transcript expression during embryogenesis, we performed RNA *in situ* hybridization on whole mount embryos (S1D–S1H Fig). *Np* transcripts are first detected during stage 11 in cells of the tracheal placodes. *Np* transcripts persist in cells of the tracheal system and also appear in the salivary glands, the foregut, the hindgut, and the posterior spiracles. During stage 16, *Np* transcripts fade in the tracheal system, while epidermal expression appears and persists during stage 17 (S1D–S1H Fig). Thus, *Np* expression during embryogenesis is specific and restricted to ectodermal tissues.

To visualize Np protein, we generated a *Np*::GFP knock-in into the endogenous *Np* gene using CRISPR/Cas9 technology (S3 Fig). Homozygous *Np*::GFP animals show no mutant phenotype and develop wild-type like until pupal stages. Np::GFP is also detectable in all tissues where the Np transcript had been found, the tracheal system, the salivary glands, the foregut, the hindgut, the posterior spiracles and the epidermis (S4 Fig). Thus, the spatial patterns of *Np* transcripts and Np::GFP protein expression coincide during embryogenesis.

The subcellular Np::GFP distribution in the tracheal system reveals intraluminal Np::GFP localization during stages 16 and 17 (Fig 1F–1G”; arrows in Fig 1F’ and 1G’). In addition, Np::GFP is also found in close association with the apical membrane of tracheal cells in stage 17 (Fig 1G–1G”; arrowheads in Fig 1G’). In the hindgut (Fig 1H–1H”) and the epidermis (Fig 1I–1I”), Np::GFP is restricted to the apical cell membranes (arrowheads in Fig 1H’ and 1I’), whereas in salivary glands it is exclusively detectable in the lumen (Fig 1J–1J”; arrow in Fig 1J’). These results indicate tissue-specific Np localizations in the aECM and/or apical cell membrane compartment. Notably, the intraluminal tracheal localization of Np::GFP is consistent with a function of *Np* in LC of the tracheal tubes.

### *Notopleural* is essential for the barrier function and apical extracellular matrix formation in tracheal tubes

**Fig 2:**
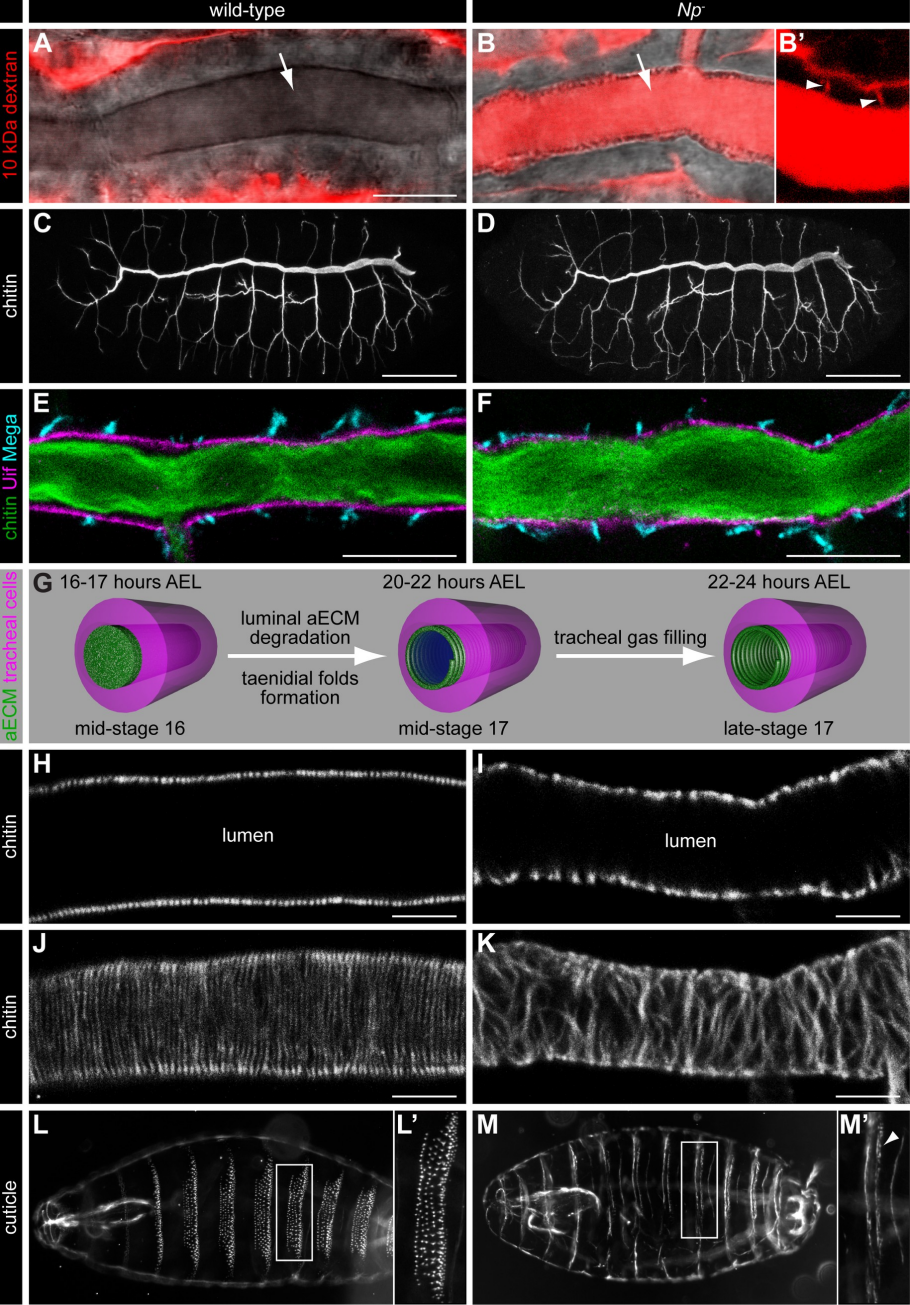
Notopleural is required for epithelial barrier function and aECM formation. (A-B’) Confocal LSM images of tracheal dorsal trunks of wild-type (A) and *Np*^*P6*^*/Np*^*C2*^ mutant (B) stage 17 embryos after Texas Red-labelled 10 kDa dextran injection into the haemocoel. Texas Red dextran (red) is not found in the dorsal trunk lumen of wild-type embryos (arrow in A), but is detectable in the dorsal trunk lumen (arrow in B) and the tracheal paracellular space (arrowheads in B’) of *Np*^*P6*^*/Np*^*C2*^ mutant embryos. (B’) shows the tracheal dorsal trunk with increased contrast to visualize the paracellular space (arrowheads in B’). (C, D) Confocal LSM images of whole-mount stainings of stage 16 wild-type (C) and *Np*^*P6*^*/Np*^*C2*^ mutant (D) embryos with FITC labelled chitin-binding probe (CBP). CBP binds the luminal chitin matrix and outlines the tracheal network during embryogenesis. The tracheal network formation of *Np* mutant embryos (D) is indistinguishable from wild-type embryos (C). (E, F) Confocal LSM images of tracheal dorsal trunks of wild-type (E) and *Np*^*P6*^*/Np*^*C2*^ mutant (F) stage 16 embryos stained with CBP (green), anti-Uif (magenta) and anti-Mega (cyan) antibodies. (G) Schema of tracheal aECM maturation and liquid clearance. The aECM (green) in the tracheal lumen is degraded after mid-stage 16 and the mature taenidial folds (green spiral) form at the apical side of tracheal cells (magenta) during mid-stage 17. Liquid (blue) is cleared from the tracheal lumen during late-stage 17. Time data refer to embryonic development at 22°C. (H-K) Confocal LSM optical sections (H, I) and sagittal z-stack projection images (J, K) of dorsal trunks of wild-type (H, J) and *Np*^*P6*^*/Np*^*C2*^ mutant (I, K) stage 17 embryos stained with CBP. (L-M’) Dark field microscopy images of stage 17 wild-type (L, L’) and *Np*^*P6*^*/Np*^*C2*^ mutant (M, M’) cuticle preparations. Denticle belts (L’, M`) develop only rudimentarily in *Np* mutant embryos (arrowhead in M’). Scale bars correspond to 10 μm in (A, B, E, F), to 50 μm in (C, D) and to 5 μm in (H-K).

Gas filling of the tracheal tubes depends on the transepithelial barrier function [32]. Since gas filling of the tracheal tubes is absent in *Np* mutants, we tested the barrier function in *Np* mutant embryos. As an assay, we injected Texas Red-labelled 10 kDa dextran into the haemocoel of stage 17 embryos [33]. Wild-type embryos show no diffusion of the dye into the lumen of the tracheal system (Fig 2A). In contrast, dextran diffuses into the tracheal lumen of *Np* mutant embryos (Fig 2B). We also observed Texas Red dextran in the paracellular space of Np mutant tracheal cells, indicating transepithelial diffusion of the dye (Fig 2B’).

This finding indicates that the tracheal barrier is lost in such embryos. Np is therefore essential for a functional transepithelial barrier in the tracheal system.

The localization of Np::GFP in the apical membrane and the aECM of tracheal cells prompted us to analyse tracheal network morphogenesis and aECM formation in *Np* mutant embryos. *Np* mutant embryos show a wild-type like tracheal tube morphology as revealed by intraluminal chitin deposition (compare Fig 2C with 2D), indicating that tracheal network morphology is not affected in *Np* mutant embryos. This finding is further supported by stainings with the cell polarity markers Uninflatable (Uif) and Crumbs, the septate junction markers Megatrachea (Mega) and Kune-kune as well as the adherens junction marker DE-cadherin. All of these marker proteins show a normal localization pattern in *Np* mutant embryos (Fig 2E and 2F; S5 Fig), indicating that cell polarity also is not affected in *Np* mutants. Also, the intraluminal chitin cable of the tracheal tubes is normally formed (Fig 2E and 2F). This fundamental element of the tubes is a transient structure and cleared from the lumen during stage 17 in both wild-type (Fig 2G and 2H) and *Np* mutant (Fig 2I) embryos. During that stage, chitin starts to form a distinct tracheal structure of the aECM, the taenidial folds [19]. These folds represent a highly regular helical structure (“chitin strands”), running perpendicular to the tube length along the lumen of the wild-type trachea (Fig 2G, 2H and 2J). We noticed that the chitin strands are disorganized in *Np* mutant embryos, i.e. strands are not arranged in parallel as observed in wild-type embryos, but are occasionally merged or fused instead (Fig 2I and 2K). Furthermore, *Np* is required for epidermal cuticle morphogenesis. Stage 17 wild-type embryos display segmentally repeated denticle belts at their ventral side that represent extensions of the epidermal aECM (Fig 2L and 2L’). In contrast, *Np* mutant embryos show impaired denticle belt formation and develop only rudimentary aECM structures on their ventral side (Fig 2M and 2M’). While these belts are also segmentally repeated, the number of denticles per belt is reduced and remaining denticles are deformed (compare Fig 2L’ and 2M’). Our results show that *Np* is required for aECM morphogenesis in the epidermis and the tracheal system of the embryo.

**Fig 3:**
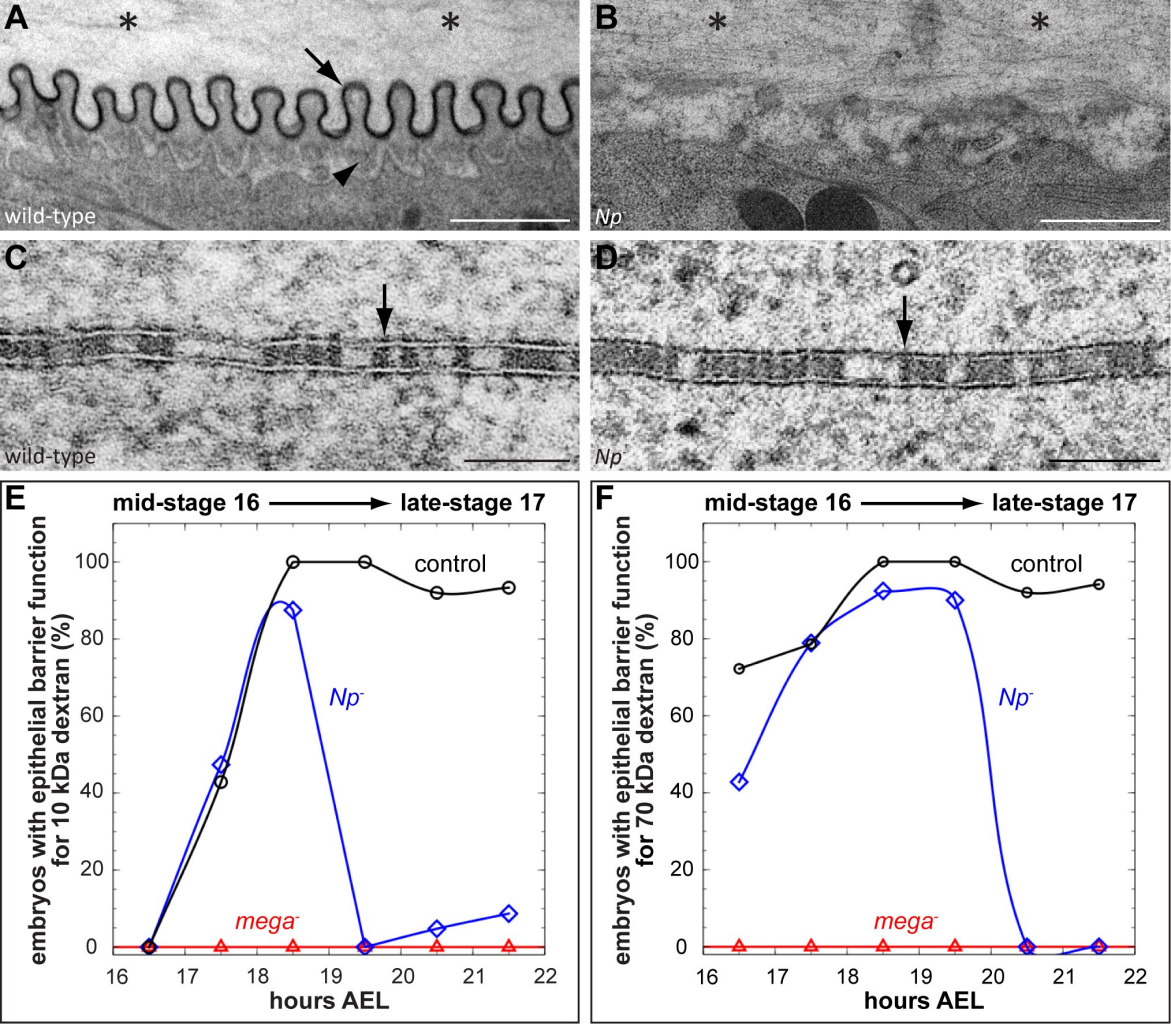
*Notopleural* mutants display defects in taenidial folds formation and maintenance of the transepithelial barrier. (A-D) Transmission electron microscopy images of stage 17 (21–22 hours AEL) wild-type (A, C) and *Np*^*P6*^*/Np*^*C2*^ mutant (B, D) tracheal aECM (A, B) and lateral tracheal cell membranes (C, D). Arrow in (A) indicates the electron-dense envelope of the taenidial folds and the arrowhead indicates the procuticle. Note the accumulation of electron-dense material in the tracheal lumen of *Np* mutant embryos (asterisks in B) as compared to wild-type embryos (asterisks in A). The ladder-like structure of SJs in wild-type (arrow in C) and *Np* mutant (arrow in D) tracheal cells is detectable. (E, F) Tracheal transepithelial barrier function proofed by 10 kDa (E) and 70 kDa (F) dextran. Dextran injections into the haemocoel of embryos reveal a tracheal barrier function (indicated by the lack of dextran diffusion into the tracheal lumen) or a defective barrier function (indicated by dextran diffusion into the tracheal system). For each indicated stage: n = 8 for *mega*; n = 15 for *Np* and control. For details see S6 Fig. Scale bars correspond to 1 μm in (A, B) and to 0.1 μm in (C, D).

In order to examine the *Np* mutant phenotype in more detail, we focused on the tracheal system by analysing the tracheal ultrastructure of *Np* mutant embryos. As shown by transmission electron microscopy, the tracheal aECM of wild-type embryos during stage 17 is characterized by evenly distributed taenidial folds (Fig 3A). They consist of the outermost electron-dense envelope (arrow in Fig 3A) and the chitin-rich procuticle (arrowhead in Fig 3A [19]). In contrast, *Np* mutant embryos develop an unstructured aECM lacking taenidial folds (Fig 3B). While such embryos contain rudimentary procuticle material, no electron-dense material, indicative of the envelope, is detectable (Fig 3B). These observations indicate that *Np* is involved in taenidial folds morphogenesis of the tracheal aECM.

### *Notopleural* is essential for the maintenance of the epithelial barrier function

Our ultrastructural analysis of *Np* mutant tracheal cells also revealed a normal wild-type ladder-like arrangement of septate junctions (SJs), the structural basis of the transepithelial barrier function (compare Fig 3C with 3D). This result was unexpected because barrier function defects were observed in *Np* mutants (see Fig 2B). Normally, such a defect correlates with damaged morphology of the SJ ultrastructure [32,34].

To analyse the barrier function defect of *Np* mutants in more detail, we injected 10 kDa and 70 kDa fluorophore-labelled dextran into the haemocoel of control, *mega*, and *Np* mutant embryos during different developmental stages (Fig 3E and 3F; S6 Fig). In control embryos, the barrier function is established during stage 16. At that stage and during stage 17, neither 10 kDa dextran nor 70 kDa dextran pass the barrier. As expected, the genuine SJ mutant *mega* lacks barrier function, since both 10 kDa and 70 kDa dye-labelled dextran diffuse into the tracheal lumen of such embryos during stages 16 and 17. In contrast, while *Np* mutants reveal wild-type-like barrier function establishment during stage 16, such embryos lack a barrier for 10 kDa dextran during early-stage 17 and subsequently, during mid-stage 17, also for the 70 kDa dextran. During late-stage 17, which is about 1 hour before LC in wild-type embryos, *Np* mutant embryos lack tracheal barrier function, similar to what has been observed in *mega* mutants (Fig 3E and 3F; S6 Fig). These results indicate that the barrier function of *Np* mutants is initially established, but it is not maintained during later stages of embryogenesis.

### Intraluminal degradation of aECM depends on *Notopleural*

**Fig 4:**
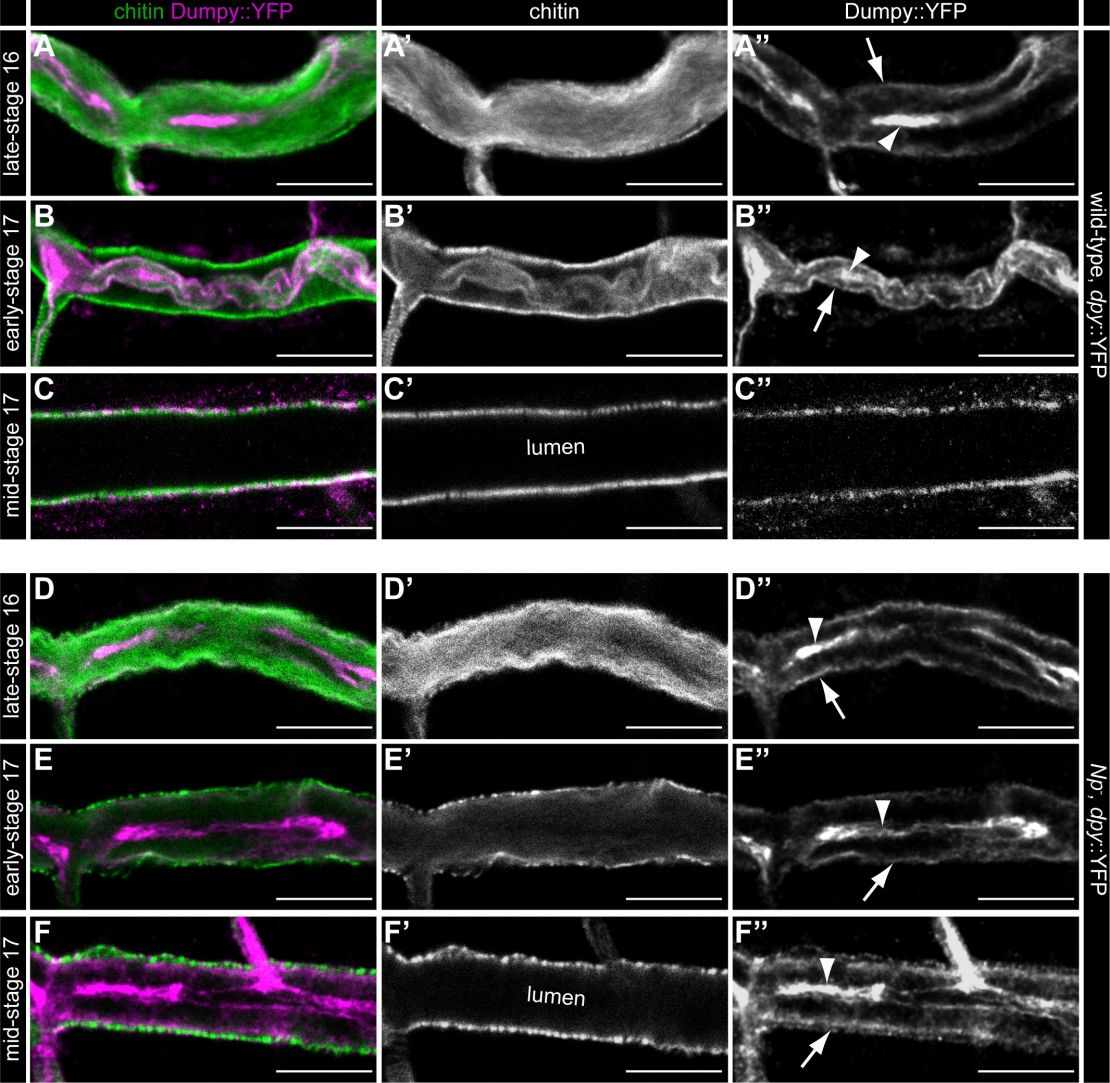
*Notopleural* is required in the trachea for luminal Dumpy degradation. Confocal LSM images of whole-mount antibody stainings of *dpy*::YFP/*dpy*::YFP (A-C”) and *Np*^*P6*^,*dpy*::YFP/ *Np*^*P6*^,*dpy*::YFP mutant (D-F”) embryos at late-stage 16 (A-A”, D-D”), early-stage 17 (B-B”, E-E”) and mid-stage 17 (C-C”, F-F”) stained with CBP and anti-GFP antibody. During late-stage 16 tracheal luminal Dpy::YFP (magenta) forms a central core (arrowhead in A”) and a peripheral “shell” layer (arrow in A”; see also [22]) in *dpy*::YFP embryos. In *Np* mutant embryos the luminal Dpy::YFP core (arrowhead in D”) and “shell” (arrow in D”) are also formed normally. Dpy::YFP and luminal chitin (green) condense at early-stage 17 (arrowhead and arrow in B”) and during mid-stage 17 the tracheal lumen is cleared from luminal chitin and Dpy (C’, C”) in *dpy*::YFP embryos. *Np* mutant embryos show no Dpy::YFP condensation during early stage 17 (arrowhead and arrow in E”; compare with B”) and no luminal clearance of Dpy::YFP during mid-stage 17 (F”; compare with C”). Note: Chitin is cleared normally from the tracheal lumen in *Np* mutant embryos during mid-stage 17 (F’) as found in *dpy*::YFP embryos (C’). Scale bars correspond to 10 μm.

When compared to wild-type embryos, we noted increased amount of electron-dense material in the tracheal lumen of *Np* mutant embryos (compare Fig 3A with 3B; asterisks indicate tracheal lumen), indicating a failure in clearance of intraluminal material. Thus, we next analysed the degradation of luminal chitin and Dumpy (Dpy), an integral component of the proteinaceous luminal matrix [3,22]. To visualize this process, we used fluorophore-conjugated chitin-binding probe and embryos that endogenously express Dpy::YFP [35]. In control embryos, the filamentous chitin matrix that is present in the tracheal lumen at late-stage 16 (Fig 4A and 4A’) condenses during early-stage 17 (Fig 4B and 4B’) and is cleared from the lumen at mid-stage 17 (asterisks in Fig 4C and 4C’). *Np* mutant embryos show a normal formation of the chitin cable (Fig 4D and 4D’) but lack the condensing process at early-stage 17 (Fig 4E and 4E’). However, such embryos clear luminal chitin normally at mid-stage 17 (Fig 4F and 4F’) as found in wild-type embryos (Fig 4C and 4C’). The Dpy::YFP cable is concentrated in a central matrix core and a peripheral region in the tracheal lumen at late-stage 16 (Fig 4A and 4A”). This luminal Dpy cable condenses similarly as found for chitin during early-stage 17 (Fig 4B and 4B”) and is cleared from the lumen during mid-stage 17 (Fig 4C and 4C”; S1 Movie). While *Np* mutant embryos form a wild-type like Dpy cable (Fig 4D and 4D”), they lack the condensation of the Dpy cable during early-stage 17 (Fig 4E and 4E”) and the luminal clearance of Dpy during mid-stage 17 (Fig 4F and 4F”; S2 Movie). These results indicate that Np activity is essential for degradation of the luminal Dpy matrix during tracheogenesis.

### Notopleural is a functional homologue of human matriptase

**Fig 5:**
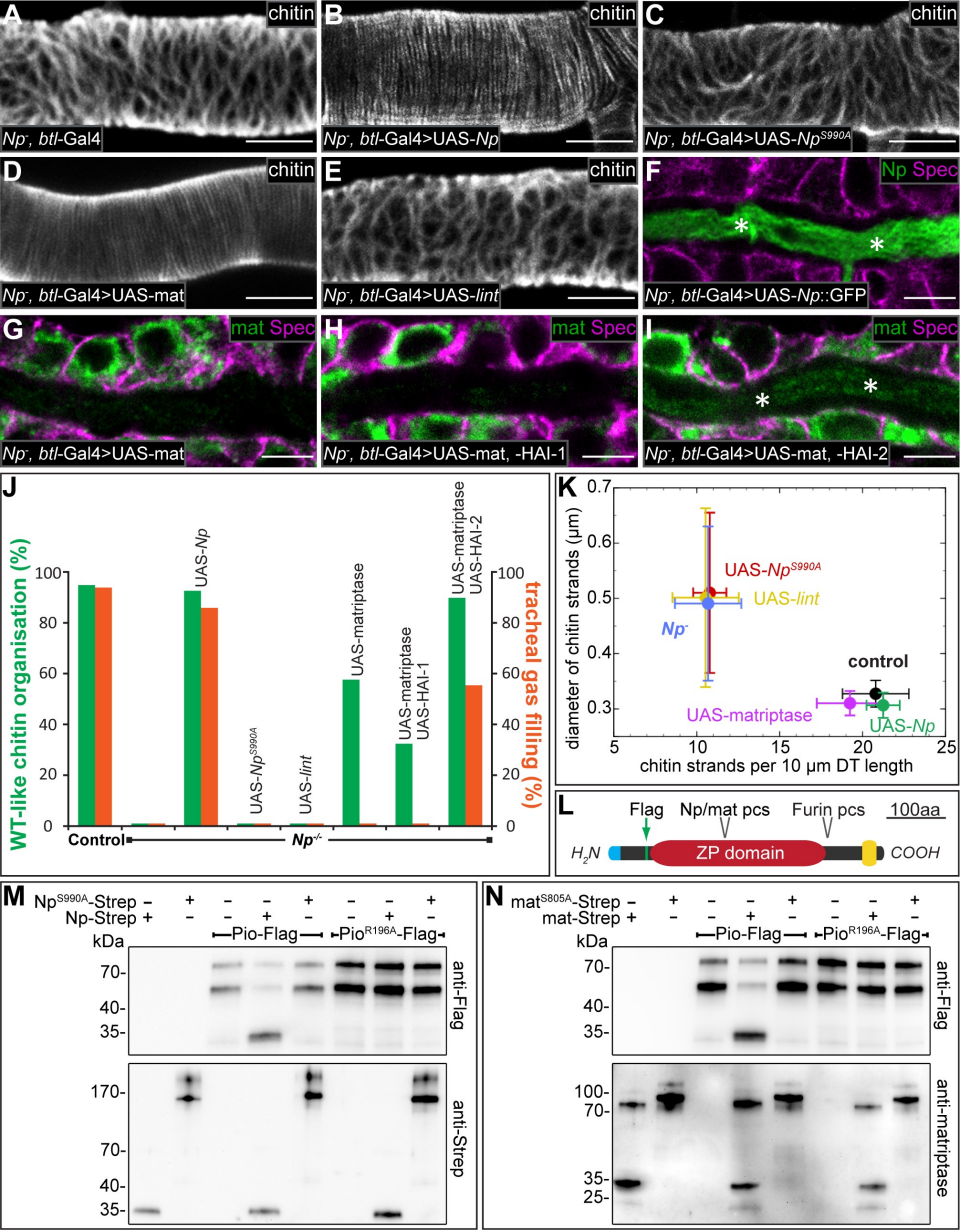
Notopleural and human matriptase are functional homologues. (A-I) Confocal LSM images of dorsal trunks of *Np*^*P6*^*/Np*^*C2*^ mutant embryos rescued by *btl-*Gal4/+ (A, control), or *btl*-Gal4/UAS-*Np*::GFP (B, F), or *btl*-Gal4/UAS-*Np*^*S990A*^ (C), or *btl*-Gal4/UAS-matriptase (D, G), or btl-Gal4/UAS-lint (E), or btl-Gal4/UAS-matriptase,UAS-HAI-1 (H), or btl-Gal4/UAS-matriptase,UAS-HAI-2 (I), stained with CBP (A-E), or anti-Spectrin (magenta) and anti-GFP (green) antibodies (F), or anti-Spectrin (magenta) and anti-matriptase (green) antibodies (G-I). Tracheal dorsal trunks of stage 17 embryos (A-E) and stage 16 embryos (F-I) are shown. Asterisks in (F) and (I) indicate matriptase (green) and Np::GFP (green) localization in the tracheal lumen. (J) Quantification of chitin matrix organisation (green bars) and tracheal gas filling (red bars). The UAS-reporter lines are driven by *btl*-Gal4 in *Np*^*P6*^*/Np*^*C2*^ mutant embryos. For each genotype: n = 120 for LC analysis; n = 40 for aECM formation (for details see Materials and Methods). (K) Quantification of number and diameter of stage 17 dorsal trunk chitin strands. Data points represent mean values for each genotype (n = 10) and error bars represent standard deviation (for details see Materials and Methods). (L) Schema showing the Piopio protein domain organization. Identified Np (see M) and matriptase (see N) protease cleavage site (pcs) is indicated. Furin pcs, signal peptide (blue), transmembrane helix (yellow) and position of Flag-tag (green) are shown. (M, N) Purified Pio-Flag or Pio^R196A^-Flag were incubated with buffer, Np-Strep, matriptase-Strep, Np^S990A^-Strep or matriptase^S805A^-Strep. Samples were analyzed by western blotting and immunostained using anti-Flag, anti-Strep or anti-matriptase antibodies. Pio-Flag is detectable in two fragments at approximately 80 and 55 kDa, presumably due to cleavage by Furin. After incubation of Pio-Flag with Np-Strep (M) or matriptase-Strep (N), a 30 kDa Pio-Flag fragment is detectable that indicates cleavage. Pio-Flag is not cleaved by catalytically inactive Np^S990A^-Strep (M) or matriptase^S805A^-Strep (N) and a single amino acid substitution in the Pio ZP domain (Pio^R196A^-Flag) is sufficient to establish cleavage resistance to Np and matriptase (M, N). Note that purified Np-Strep (M) and matriptase-Strep (N) are detected as approximately 35 kDa fragments, consistent with the predicted sizes of the catalytic protease domains and, thus, indicating zymogen activation. Purified catalytically inactive Np^S990A^-Strep (M) and matriptase^S805A^-Strep (N), are detected as 150–200 kDa and 90–120 kDa zymogens, respectively. These results indicate autocatalytic zymogen activation for both proteases. Scale bars correspond to 5 μm.

The sequence homology of Np and human matriptase (see S2 Fig) and the similar roles of both proteins in aECM formation and in epithelial barrier function suggest that matriptase and Np are functionally homologous. To test this possibility, we performed rescue experiments of the *Np* mutation using the UAS/Gal4 system (Fig 5A–5K). Pan-tracheal expression of Np by the *btl-*Gal4 driver rescues the *Np* phenotype of tracheal chitin malformation (compare Fig 5A and 5B; see also 5J). To examine whether Np indeed carries protease activity, we generated Np^S990A^, which contains a single amino-acid mutation in the catalytic triad known to disrupt the activity of serine proteases [36]. Tracheal expression of Np^S990A^ in *Np* mutants is insufficient to rescue chitin malformation (Fig 5C, see also 5J), indicating the requisite of *Np* catalytic protease activity for *Np* function. Furthermore, tracheal expression of human matriptase in *Np* mutants also rescues the chitin malformation (Fig 5D), similar to what has been observed with *Drosophila* Np (Fig 5B; see also 5J).

To elucidate the specificity of matriptase activity, we performed rescue experiments using the TTSP Lumens interrupted (Lint), which is encoded 5 kb downstream of *Np* and may act in the processing of luminal tracheal matrix proteins [37]. The catalytic domains of Lint and Np share high sequence identity of 44% (S7 Fig). However, tracheal expression of Lint in *Np* mutant embryos fails to rescue the *Np* chitin malformation (Fig 5E and 5J).

These observations were verified by qualitative analysis of chitin aECM formation (Fig 5K). UAS-matriptase and UAS-*Np* rescued *Np* mutants as well as control (wild-type) embryos have about 20 chitin strands per 10 μm dorsal trunk length and the diameter of the dorsal trunk chitin strands is about 0.3 μm. In contrast, *Np* mutants as well as UAS-*Np*^*S990A*^ (catalytically inactive protease) and UAS-*lint* rescued *Np* mutants exhibit only 10 chitin strands per 10 μm dorsal trunk length and the diameter of the dorsal trunk chitin strands is about 0.6 μm (Fig 5K). These results imply a target-specific functional similarity of Np and matriptase beyond general protease activity. Notably however, tracheal expression of Np results in wild-type-like gas filling of the *Np* mutant tracheal system (Fig 5J), while the corresponding matriptase expression does not mediate tracheal gas filling of *Np* mutant embryos (Fig 5J). Also, while ectopically expressed Np is localized normally in the tracheal lumen (asterisks in Fig 5F), matriptase expression results in an intracellular localization (Fig 5G). In vertebrates, matriptase processing involves a transient interaction with its cognate cofactors, hepatocyte growth factor activator inhibitor (HAI) -1 and -2 [8,38]. Thus, we analysed the effects of human HAI-1 and HAI-2 in *Np* mutant embryos expressing human matriptase. Coexpression of matriptase and HAI-1 had no effect on matriptase localization, i.e. matriptase retains its intracellular localization (Fig 5H). Such embryos lack tracheal gas filling (Fig 5J). In contrast, coexpression of matriptase and HAI-2 in *Np* mutant embryos reveals luminal matriptase localization (asterisks in Fig 5I) and gas filling of the tracheal system (Fig 5J), i.e. the *Np* mutant phenotype was rescued as observed with *Np* expression. These results demonstrate that luminal matriptase facilitates LC of the tracheal system in the *Np* mutant. Furthermore, ectopic tracheal Np or matriptase expression in *Np* mutants mediates degradation of Dpy::YFP as observed in wild-type trachea, whereas catalytic-inactive Np^S990A^ expression does not degrade Dpy::YFP (S8 Fig). In summary, our rescue experiments suggest that *Drosophila* Np is a functional homologue of human matriptase, which, however, is secreted *via* a different mechanism.

The observation that matriptase mediates degradation of Dpy was puzzling since Dpy is not conserved in vertebrates. However, Dpy contains a ZP domain, which is conserved in many invertebrate and vertebrate ECM proteins [39]. Since Dpy is a 2.5 MDa large and highly modified protein [40], we chose the ZP-domain protein Pio to study putative ZP-domain cleavage *in vitro*. Pio co-localizes with Dpy in the tracheal lumen. The two proteins display similar functions and they possibly interact *via* their ZP domains [41]. To test Np protease activity *in vitro*, we expressed full-length C-terminally Strep-tagged Np (Np-Strep) in *Drosophila* Kc cells. Purification followed by SDS polyacrylamide gels and anti-Strep immunoblots show the expression of an approximately 32 kDa Strep-immunoreactive protein (Fig 5M). Thus, purified recombinant Np yields a protein with an apparent molecular mass of the Np catalytic protease domain, implying that the protein is cleaved for activation. For control, we also purified catalytically inactive Np^S990A^-Strep. With this mutant protein, no cleavage is observed, as evident from the absence of the 32 kDa catalytic domain (Fig 5M). These results indicate autoactivation of Np, presumably *via* the interaction of Np zymogens as has been described for matriptase activation [14]. Furthermore, *in vitro* assays combining full-length Flag-tagged Pio (Pio-Flag; Fig 5L) and Np-Strep result in a cleaved Flag-tagged Pio fragment of approximately 30kDa, while inactive Np^S990A^-Strep lacks the capability to cleave Pio-Flag (Fig 5M). This shows that Np protease cleaves Pio *in vitro*. The size of the fragment implies cleavage within the ZP domain of Pio. We also purified functional matriptase (mat-Strep) as done for Np and analysed its ability to cleave Pio-Flag (Fig 5N). Mat-Strep generates a Pio-Flag cleavage product as observed with Np protease (Fig 5M and 5N). To identify the Np and matriptase target cleavage site in Pio-Flag, we generated eight single point mutations within the protease cleavage site region of Pio-Flag. One of them, Pio^R196A^-Flag is cleavage-resistant to both Np and matriptase (Fig 5M and 5N). These results show that Np and matriptase cleave the Pio ZP domain at similar or identical positions, indicating that the conserved ZP domain contains the target cleavage site for Np and matriptase, respectively.

### The matriptase target prostasin is conserved in *Drosophila*

**Fig 6:**
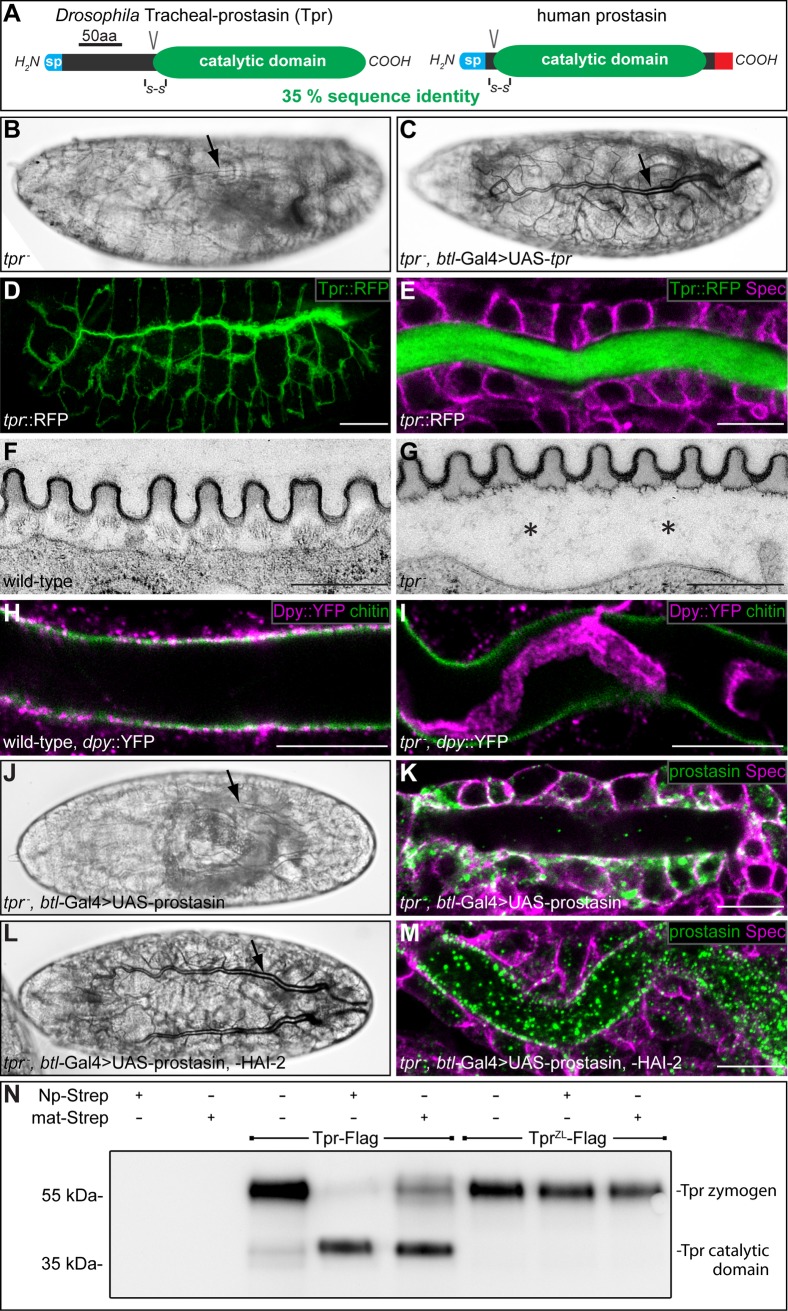
*Drosophila* Tracheal-prostasin and human prostasin show functional similarity. (A) Schema showing the protein domain organization of *Drosophila* Tpr and human prostasin. The signal peptides (sp; blue), conserved disulfide bridges (S-S), the activation cleavage sites (V), the catalytic protease domains (green) and the GPI anchor (red) are indicated. (B, C) Bright field light microscopic images of stage 17 *tpr*^*D1*^*/tpr*^*D1*^ (B) and *tpr*^*D1*^,*btl*-Gal4/*tpr*^*D1*^,UAS-*tpr* (C) mutant embryos. (D, E) Whole-mount antibody stainings of stage 16 *tpr*::RFP embryos stained with anti-RFP (D, E) and anti-Spectrin (E) antibodies. Tpr::RFP (green) is restricted to the tracheal system (D) and localized in the tracheal lumen (E). Spectrin (magenta) outlines the tracheal cells (E). (F, G) Transmission electron microscopic images of stage 17 wild-type (F) and *tpr*^*D1*^*/tpr*^*D1*^ mutant (G) tracheal aECM. The taenidial folds of the tracheal aECM are associated with the apical side of tracheal cells in wild-type (F), while the taenidial folds are detached from tracheal cells in *tpr* mutant embryos (asterisks in G). (H, I) Whole-mount antibody stainings of *dpy*::YFP/*dpy*::YFP (H) and *tpr*^*D1*^,*dpy*::YFP/*tpr*^*D1*^,*dpy*::YFP mutant (I) embryos at stage 17 stained with FITC labelled CBP (green) and anti-GFP antibodies (magenta). Chitin is cleared from the tracheal lumen of wild-type (H) and *tpr* mutant (I) embryos. Dpy::YFP is cleared from the tracheal lumen of wild-type embryos (H), while Dpy::YFP degradation is incomplete in *tpr* mutant embryos (I). (J-M) Bright field light microscopic images of stage 17 (J, L) and confocal LSM images of whole mount antibody stainings of stage 16 (K, M) *tpr*^*D1*^,*btl*-Gal4/*tpr*^*D1*^,UAS-prostasin (J, K) and *tpr*^*D1*^,*btl*-Gal4/*tpr*^*D1*^,UAS-prostasin; UAS-HAI-2/+ (L, M) mutant embryos with anti-prostasin (green) and anti-Spectrin (magenta) antibodies. *tpr* mutant embryos expressing prostasin in the trachea lack tracheal gas filling (arrow in J) and show prostasin localization in tracheal cells (green in K). In contrast, tracheal co-expression of prostasin and HAI-2 in *tpr* mutant embryos facilitates gas filling of the tracheal system (arrow in L) and prostasin localizes to the tracheal lumen (green in M). (N) Purified Tpr-Flag or zymogen locked Tpr^ZL^-Flag were incubated with buffer, Np-Strep or matriptase-Strep and samples were analysed by western blotting and immunostained using anti-Flag antibody. Tpr-Flag is detectable as a fragment of approximately 60 kDa that is processed to a fragment of approximately 40 kDa by incubation with purified Np-Strep or purified matriptase-Strep. Proteolytic processing occurs at the zymogen activation site, since zymogen locked Tpr^ZL^-Flag is resistant to cleavage by Np-Strep and matriptase-Strep. Scale bars correspond to 1 μm in (F, G) to 10 μm in (E, H, I, K, M) and to 50 μm in (D).

In humans, matriptase is an efficient zymogen activator of the serine protease prostasin. Matriptase and prostasin are coexpressed in overlapping spatial and temporal patterns. Mice deficient in epidermal prostasin display defects that are identical to matriptase-deficient mice. It was postulated that matriptase acts *via* prostasin as an initiator of a proteolytic cascade in the epidermis [8]. We investigated whether the matriptase-prostasin proteolytic cascade is also conserved in *Drosophila*. *In silico* analysis identified by sequence homology to prostasin a putative serine protease of *Drosophila* encoded by *CG4386*, to which we refer as *tracheal-prostasin (tpr*; Fig 6A, S9 Fig).

Visualization of the *tpr* mRNA by *in situ* hybridization of embryos shows *tpr* expression exclusively in the tracheal system from stage 15 to stage 17 (S10A–S10D Fig). Thus, Np expression precedes *tpr* expression in the tracheal system. This finding is consistent with the conclusion that the Tpr serine protease is a target of Np. For phenotypic analysis, we performed pan-tracheal RNAi-mediated *tpr* knockdown (*btl*-Gal4; UAS-RNAi-*GD5850* [29]) and generated loss-of-function *tpr* alleles by point mutations (*tpr*^*D1*^ and *tpr*^*F1*^) and a specific *tpr* deletion (*Δtpr-attP*) using the CRISPR/Cas9 system (S10E–S10G Fig; S11 Fig). RNAi-mediated knockdown embryos, homozygous *tpr*^*D1*^and *tpr*^*F1*^ mutant embryos lack tracheal LC and die during the first instar larval stage (Fig 6B). Pan-tracheal *tpr* expression in *tpr* mutant embryos using the UAS/Gal4 system rescues the LC phenotype (Fig 6C) and the embryos develop to adult and fertile flies. These findings indicate that *tpr* function is restricted to tracheal development and is essential for normal gas filling of the tracheal system as observed with *Np*.

To visualize Tpr protein in the *Drosophila* embryo, we generated a *tpr*::RFP knock-in into the endogenous *tpr* gene (S11 Fig). Homozygous *tpr*::RFP animals develop to normal fertile flies. Tpr::RFP is exclusively detectable in the tracheal system during stage 16 of embryogenesis (Fig 6D). Tpr::RFP remains in the trachea and is almost exclusively found in the tracheal lumen (Fig 6E). This finding prompted us to analyse the tracheal aECM formation of *tpr* mutant embryos in more detail. Ultrastructural analysis of the *tpr* mutant tracheal aECM by transmission electron microscopy revealed that in *tpr* mutant embryos the taenidial folds are detached from the apical side of tracheal cells (asterisks in Fig 6G compare with wild-type in 6F). However, taenidial folds formation is not affected in *tpr* mutant embryos (Fig 6G). While luminal Dpy::YFP is cleared in wild-type embryos during mid-stage 17 (Fig 6H), Dpy::YFP degradation is incomplete in *tpr* mutant embryos, which still display rudiments of Dpy::YFP in the tracheal lumen (Fig 6I). These results indicate that tracheal aECM formation and degradation of tracheal luminal Dpy is affected in *tpr* mutant embryos.

The rescue of the *tpr* LC phenotype by pan-tracheal *tpr* expression (see Fig 6C) prompted us to test the rescuing activity of human prostasin as well. No rescue of the LC phenotype can be observed by pan-tracheal expression of human prostasin in *tpr* mutant embryos (Fig 6J). In such embryos, however, human prostasin is predominantly localized in the cytoplasm of tracheal cells (Fig 6K), whereas *Drosophila* Tpr is localized in the tracheal lumen (see Fig 6E). This finding suggests that human prostasin is not properly processed in *Drosophila* cells. Thus, we co-expressed human prostasin along with the human co-factors HAI-1 or HAI-2. While HAI-1 co-expression showed no LC rescue, we observed rescuing activity of human prostasin after co-expression with HAI-2 in *tpr* mutants, i.e. the embryos show normal tracheal LC (Fig 6L). In such embryos, human prostasin is localized exclusively in the tracheal lumen (Fig 6M), indicating exocytosis of prostasin *via* HAI-2. These results demonstrate that *Drosophila* Tpr and human prostasin are functional homologues as has been observed for Np and human matriptase.

### Tracheal-prostasin is cleaved by Notopleural and matriptase

The functional homology of Tpr and human prostasin suggests that Np could be a physiological activator of Tpr zymogen. To test this proposal, we performed *in vitro* experiments using C-terminally Flag-tagged Tpr (Tpr-Flag; Fig 6N). The Flag-tagged Tpr appears to be predominantly in the zymogen form, as suggested by its size of 55 kDa. For control, we generated Flag-tagged zymogen-locked Tpr (Tpr^ZL^-Flag), which contains mutations in the putative cleavage site for zymogen activation. *In vitro* exposure of Tpr-Flag to Np-Strep results in cleaved Tpr zymogen, while Tpr^ZL^-Flag is not cleaved under the same conditions. Also, matriptase cleaves Tpr-Flag but not Tpr^ZL^-Flag (Fig 6N). These *in vitro* results indicate that both Np and matriptase cleave Tpr at the putative zymogen activation site and suggest that Tpr is also a direct target of Np in the embryo.

## Discussion

We report that the vertebrate matriptase-prostasin proteolytic cascade, which is crucial for extracellular matrix differentiation and tissue homeostasis, is conserved in *Drosophila*. Np acts as a functional *Drosophila* homologue of matriptase, and *tpr* mediates prostasin function in the *Drosophila* tracheal system. Cleavage targets of these conserved extracellular proteolytic pathways are the ZP domains, present in many extracellular proteins of both vertebrates and invertebrates. The Np-mediated protease cascade controls at least three distinct cellular processes during tracheal development, i.e. morphogenesis of the taenidial folds, degradation of the tracheal Dpy cable in the tracheal lumen, and maintenance of the transepithelial barrier function.

### Conserved protease cascades mediate epithelial differentiation

In vertebrates, proteolysis by matriptase plays a key role in regulating epithelial differentiation. Ectopic expression of human matriptase in the developing tracheal system of *Np* mutant embryos rescues all aspects of the *Np* mutant phenotype, i.e. degradation of the Dpy luminal cable, taenidial folds formation, and gas filling of the tubes. The fact that matriptase can functionally substitute for the lack of Np activity indicates that the two proteins share similar functions. Furthermore, similar to matriptase, Np is differentially localized in tissue and stage-dependent patterns at the apical plasma membrane and the apical extracellular space. These findings suggest processing of Np by ectodomain shedding similar to what has been described for matriptase [42]. However, the processing of human matriptase involves not only ectodomain shedding but also the transient interaction of the stem region with its cognate inhibitor HAI-2 [43]. Absence of HAI-2 prevents cell surface translocation of matriptase and causes its accumulation in the Golgi compartment [38]. Ectopic HAI-2 expression in combination with matriptase facilitates secretion of matriptase in the *Drosophila* tracheal system. This finding and the lack of a *Drosophila* HAI-2 homologue indicate different regulatory mechanisms for the translocation and ectodomain shedding of Np and matriptase. This assumption is also consistent with the lack of conserved regulatory stem regions of Np and matriptase. Therefore, we propose different protein processing mechanisms of the two otherwise functionally equivalent proteins. However, the apparent diverse regulation of both proteins provides the possibility to establish an *in vivo* experimental system to analyse aspects of matriptase regulation and processing in *Drosophila*.

The matriptase-prostasin proteolytic cascade is initiated by rapid matriptase autoactivation as shown by an *in vitro* cell-free system [14] as we observed with Np. Thus, the proteolytic cascade involved in aECM formation and maturation in *Drosophila* is likely to be initiated by Np autoactivation. Once activated, it acts on Tpr, a direct downstream target of Np in the developing tracheal system. This conclusion is based on the observation that *in vitro-*purified Np is able to cleave Tpr at the zymogen activation site, implying a direct activation of Tpr zymogen by Np *in vivo*. Furthermore, the tracheal aECM phenotype of *tpr* mutant embryos is less pronounced than the *Np* phenotype, since taenidial folds are wild-type like in *tpr* mutant embryos, while Np mutant embryos lack taenidial folds. The observation that human matriptase also cleaves Tpr at the same zymogen activation site provides additional support for the functional identity of matriptase and Np.

Ectopic tracheal expression of human prostasin, together with human HAI-2 in *tpr* mutant embryos, rescues the defects of the aECM and the LC phenotype of *tpr* mutants. Thus, Tpr is a functional homologue of human prostasin in the developing trachea. Also, human HAI-2 is required for prostasin secretion into the tracheal lumen, as has been reported for prostasin in vertebrate tissues [43]. LC defects, as observed in *tpr* mutants, are often caused by an impaired transepithelial barrier function. However, *tpr* mutant embryos develop a normal barrier and, thus, we suppose that the LC defects are likely caused by hampered degradation of luminal material and/or Tpr might affect epithelial sodium channels (ENaCs). ENaCs are located in the apical membrane of tracheal cells and are critical for tracheal gas filling [44]. In vertebrates, prostasin activates ENaCs by inducing proteolytic cleavage of the gamma subunit [45]. It will be interesting to see whether Tpr plays a similar role for ENaC activation in *Drosophila*.

Vertebrate prostasin is widely expressed in ectodermal tissue and most functional aspects of human matriptase are mediated *via* prostasin in the various tissues [8]. However, the functional relationship of matriptase and prostasin remains to be clarified since matriptase activation and shedding is prostasin-dependent in specific tissues [43,46]. In contrast to vertebrate prostasin, *Drosophila* Tpr expression is confined to the tracheal system and not detectable in other ectodermal tissues. Thus, while vertebrate matriptase and prostasin are co-expressed in most tissues we propose a different scenario in *Drosophila*. Tpr belongs to a small group of seven very similar proteases in *Drosophila* [47]. The corresponding genes are differentially expressed in specific spatial patterns in various ectodermal tissues [48]. We propose that such protease zymogens represent putative Np cleavage targets that mediate Tpr-like functions in the different ectodermal tissues that express Np including salivary glands, hindgut, and epidermis.

### The role of Notopleural and Tracheal-prostasin during tracheal organ morphogenesis

*Drosophila* tracheal development is a paradigm for the generation of branched tubular systems [49]. Early steps of tracheal maturation, notably tube formation and tubular network assembly, develop independently of *Np*, while tracheal aECM formation and the transepithelial barrier function during late embryonic tracheogenesis depend on *Np*. Main differentiation events of the aECM, such as taenidial folds morphogenesis and degradation of luminal protein matrix, are controlled by *Np* and *Tpr*.

The taenidial folds of the tracheal aECM mainly consist of chitin running perpendicular to the tracheal tube length along the lumen. Their main function is to provide stiffness combined with concurrent flexibility of the tube [19]. Taenidial folds formation is severely affected in *Np* mutant embryos. The outmost taenidial structure, the hydrophobic envelope, is not detectable and the chitin strands of taenidial folds are highly disorganized. Chitin-interacting proteins, involved in chitin organization, may represent putative targets of Np activity. The aECM phenotype of *tpr* mutant embryos suggests that *tpr* function is more specific and confined to establish a proper adhesion between the apical side of tracheal cells and the overlaying taenidial folds of the aECM.

Both Np and Tpr are also involved in the degradation of tracheal luminal Dpy, a large ZP domain-containing protein. Luminal Dpy is part of a chitin-proteinous matrix within the tracheal lumen and is essential for normal tracheal network and tube formation [41]. The luminal matrix is degraded and removed from the tracheal lumen during formation of the tracheal taenidial folds and the subsequent gas filling of the tracheal system. Np appears to be the key factor in Dpy cable degradation since *Np*-deficient embryos completely lack Dpy degradation. Some degradation, however, is mediated *via* Tpr proteolysis because *tpr* mutants display remaining Dpy material in the tracheal lumen during late embryogenesis. Thus, the combination of Np and Tpr accomplish luminal Dpy degradation. Alternatively or in addition, Np activates unknown proteases that mediate complete Dpy degradation prior to the gas filling of the tracheal tubes.

### Notopleural controls maintenance of the epithelial barrier function

The transepithelial barrier function is established by the septate junction (SJ) protein complexes, localized at the apico-lateral membrane of epithelial cells [50]. The lack of *bona fide* SJ proteins like the *Drosophila* claudin Mega causes a disruption of the ladder-like SJ ultrastructure and a barrier function defect [32]. In *Np* mutants, the ladder-like ultrastructure of SJs and the barrier function appear to be properly established, but the barrier function collapses during the end of embryogenesis. Thus, Np is essential for the maintenance of the transepithelial barrier function mediated by SJs. This function of Np is reminiscent of matriptase function in mammals. Tracer injection experiments into the dermis of matriptase-deficient mice indicate impaired epidermal tight junction function in such animals [18]. In intestinal epithelial model cell layers and hypomorphic matriptase mice, the essential tight junction component Claudin-2 is deregulated. This observation suggests that reduced barrier integrity was caused, at least in part, by an impaired claudin-2 protein turnover [51]. Furthermore, matriptase cleaves EpCAM, which in turn decreases EpCAM ability to associate with claudin-7 followed by lysosomal degradation of claudin-7 [52]. Thus, we speculate that Np may also control the maintenance of the epithelial barrier in *Drosophila* by regulating the function or turnover of claudins in SJs, the invertebrate analogue of the vertebrate tight junction.

### ZP-domain proteins represent putative targets of Notopleural and matriptase

The aECM protein Dpy is an *in vivo* downstream target of both Np and human matriptase proteolytic activity. This observation was puzzling since Dpy is not conserved in vertebrates [53]. However, Dpy contains a conserved region, the ZP domain [39]. The ZP domain defines a conserved family of aECM proteins, originally identified in the zona pellucida coat surrounding the mammalian oocyte [54]. The 260 amino acids long ZP domain is proposed to act as a module promoting polymerization of proteins into threads and matrices essential for the organization of highly specialized apical extracellular structures [55]. In fact, the ZP domain was confirmed as a target of Np and matriptase by our data showing that Pio, an aECM protein containing a ZP domain [56], was cleaved by Np and matriptase. Both cleave the ZP domain of Pio within the short linker region, which separates ZP-N and ZP-C, the two subdomains of the ZP domain. Pio is secreted apically in the tracheal lumen and establishes together with Dpy, possibly *via* ZP-domain polymerization, a structural matrix in the tracheal lumen that is essential for the formation of an interconnected branched network. Cleavage of ZP domains within a meshwork of Dpy and Pio filaments may facilitate rapid degradation of the luminal extracellular matrix, the prerequisite for normal gas filling of the tracheal system. This conclusion is supported by phenotypic analysis of Np mutant embryos, which exhibit a stable, undegraded luminal Dpy cable and lack tracheal gas filling. ZP-domain proteins also play crucial roles in development of embryonic epidermal cuticle, an aECM that protects the animal against the external milieu [57,58]. Eight ZP-domain proteins are required for the localized reorganization of epidermal cells and to sculpture the actin-rich apical extensions, the denticles [58]. Our observation that Np mutants exhibit reduced and rudimentary denticles in the epidermis underlines the possibility that the epidermal ZP-proteins are also targets of Np protease activity.

Our results showing that human matriptase cleaves the ZP domain of Pio open future directions to explore novel targets of the matriptase-prostasin catalytic pathway. In vertebrates, ZP-domain proteins are involved in remodelling apical extracellular structures, such as ZP1-ZP3, important in the mammalian ovary for fertilization [59] and uromodulin, which is released into the tubular kidney lumen where it polymerizes in a gel-like matrix that controls salt transport and urine concentration [60]. Also, mutations in genes encoding ZP-domain proteins cause human diseases such as deafness, triggered by mutations in alpha- and beta-tectorin. The tectorins are components of the tectorial membrane, an aECM necessary for sound transmission to neural cells in the cochlea [61]. These examples already demonstrate the importance of ZP-domain proteins for mammalian physiology. Based on the results reported here, we propose that ZP-domain cleavage by the matriptase-prostasin proteolytic cascade may represent a conserved process to control ZP-domain protein functions, which are crucial for apical matrix remodelling during development, wound repair, and differentiation.

## Materials and methods

### *Drosophila melanogaster* strains and genetics

Flies were kept at 22°C using standard procedures and used strains are listed in Table 1. Loss-of-function mutants *Np*^*P6*^, *Np*^*C2*^, *tpr*^*D1*^, *and tpr*^*F1*^ were established for this study using CRISPR/Cas9-mediated mutagenesis as described in S1 Fig and S10 Fig. The *Np*::GFP and *tpr*::RFP strains were established for this study, exploiting CRISPR/Cas9-mediated homology directed repair (HDR) and ΦC31 integrase-mediated transgenesis [62] as described in S3 Fig and S11 Fig. Target sequences of sgRNAs were cloned into *pBFv-U6*.*2* vector [63]. Sequences of homology arms (HAs) were amplified by PCR from genomic DNA of *nos-Cas9-3A* flies (National Institute of Genetics, Mishima, Japan; stock: CAS-0003). *Np* HAs were cloned into *pGX-attP* vector [64], and *tpr* HAs were cloned into *pHD-DsRed-attP* vector [62]. Vectors for sgRNA expression and HDR template vectors were injected into *nos-Cas9-3A* embryos by BestGene Inc. (Chino Hills, CA, USA). For ΦC31 integrase-mediated knock in of *Np*::GFP and *tpr*::*RFP* rescue constructs, corresponding genomic regions were amplified by PCR from *w*^*1118*^ genomic DNA, using primers to attach restriction sites for cloning into *pGE-attB-GMR* vector [64] and to delete the endogenous stop codons and attach restrictions sites at the ORF 3' end to subsequently add fluorophore-encoding sequences.

**Table 1:** *Drosophila melanogaster* strains.

*D*. *melanogaster* strains	Source	Identifier
*btl*-Gal4: *w*^(*)^*; btl-Gal4*	Ohshiro and Saigo, 1997	N/A
RNAi of *Np (CG34350)*: *w*^*1118*^*;P{GD13443}v23381/TM3*	VDRC	Cat# 23381; RRID: FlyBase_FBst0454979
*Np*^*P6*^ mutant allele: *w*^(*)^*; Np*^*P6*^*/CyO*,*dfd-eYFP*	This study	N/A
*Np*^*C2*^ mutant allele: *w*^(*)^*; Np*^*C2*^*/CyO*,*dfd-eYFP*	This study	N/A
Np overexpression: *w*^(*)^*;;P{UAS-Np}3*	This study	N/A
*Np*::eGFP: *w*^(*)^*; Np*^*attL*,*eGFP*^*/CyO*,*dfd-eYFP*	This study	N/A
*mega*^*VE896*^ mutant allele: *mega*^*VE896*^*/FM7a*	BDSC	Cat# 4645; RRID: BDSC_4645
*dumpy*::YFP: *w*^*1118*^*; PBac{681*.*P*.*FSVS-1}dpy[CPTI001769]*	DGGR (Kyoto)	Cat# 115238; RRID: DGGR_115238
Np^S990A^ overexpression: *w*^(*)^*;;P{UAS-Np*^*S990A*^*}3*	This study	N/A
Lint overexpression: *w*^(*)^,*P{UAS-lint}1*	This study	N/A
Human matriptase overexpression: *w*^(*)^*;;P{UAS-hmatriptase}3*	This study	N/A
Human HAI-1 overexpression: *w*^(*)^*;;P{UAS-hHAI-1}3*	This study	N/A
Human HAI-2 overexpression: *w*^(*)^*;;P{UAS-hHAI-2}3*	This study	N/A
RNAi of *tpr (CG4386)*: *w*^(*)^*;P{KK107228}VIE-260B*	VDRC	Cat# 109488; RRID: FlyBase_FBst0481175
*tpr*^*D1*^ mutant allele: *w*^(*)^*;tpr*^*D1*^*/CyO*,*dfd-eYFP*	This study	N/A
*tpr*^*F1*^ mutant allele: *w*^(*)^*;tpr*^*F1*^*/CyO*,*dfd-eYFP*	This study	N/A
Tpr overexpression: *w*^(*)^*;;P{UAS-tpr}3*	This study	N/A
*tpr*::mCherry: *w*^(*)^*; tpr*^*attL*,*mCherry*^*/CyO*,*dfd-eYFP*	This study	N/A
Human prostasin overexpression: *w*^(*)^*;P{UAS-hprostasin}2*	This study	N/A

The *btl-*Gal4 strain [65] was used to drive expression of UAS-*RNAi-GD13443* (VDRC ID23381) and UAS-*RNAi-KK107228* (VDRC ID109488) for pan-tracheal RNAi-mediated knockdowns of *Np* and *tpr*. The *dpy*::YFP strain (Kyoto DGRC, stock: 115238) was used for analysis of Dpy localization. The *mega*^*VE896*^ mutant (BDSC ID 4645) was used as a model for lack of tracheal epithelial barrier function.

For tracheal system-specific rescue experiments with *btl-*Gal4 driver, the following UAS-responder lines were established by amplification of ORF-encoding sequences using PCR, subsequent cloning into *pUAST* vector [28] and P-element-mediated germline transformation [66]: UAS-*Np*, UAS-*Np*::GFP, UAS-*Np*^*S990A*^, UAS-*lint*, UAS-human-matriptase, UAS-human-HAI-1, UAS-human-HAI-2, UAS-*tpr*, and UAS-human-prostasin. ORF amplification of *Np* was done from cDNA that was generated from purified total embryonic RNA using Superscript IV kit (Thermo Fisher Scientific, Cat# 18091050). ORF amplifications of *tpr* and *lint* were done from plasmids LD47230 and LD43328 respectively (DGRC). Plasmid SC125523 (OriGene) was the template for human matriptase ORF, and plasmid SC118401 (OriGene) was the template for human prostasin ORF. Human HAI-1 and HAI-2 ORF sequences were synthesized (MWG, Eurofins genomics) with codon optimization for *Drosophila* according to cDNA sequences AY358969 and BC001668 respectively (PubMed; S12 Fig).

### Immunohistochemistry

Whole-mount immunostainings of fixed embryos were performed as described previously [67]. The following primary antibodies were used: chicken anti-GFP (1:500; Abcam), mouse anti-Spectrin 3A9 (1:10; DSHB), guinea pig anti-Uif (1:500; [68]), mouse anti-Mega (1:20; [69]), mouse anti-Crumbs Cq4 (1:50; DSHB), rat anti-DE-cadherin DCAD2 (1:50; DSHB), sheep anti-human-matriptase (1:250; R&D Systems), rabbit anti-human-prostasin (1:250; GeneTex), rabbit anti-human-HAI-2 (1:200; ThermoFisher), and rabbit anti-mCherry (1:500; Rockland). The following secondary antibodies were used in 1:500 dilutions: goat anti-mouse IgG Alexa568, goat anti-mouse IgG Alexa488, goat anti-guinea pig IgG Alexa488, goat anti-rabbit IgG Alexa568, goat anti-rabbit IgG Alexa488 (Invitrogen), goat anti-chicken Alexa488 (Jackson Immuno Research), donkey anti-chicken DyLight549 (Jackson Immuno Research), and donkey anti-sheep Alexa568 (Invitrogen). Fluorescein-conjugated chitin-binding probe (NEB) was used in a 1:500 dilution to stain chitin. Stained embryos were mounted in ProLong Gold antifade reagent (Invitrogen). Image acquisitions were performed with a LSM780 confocal microscope (Zeiss) and a LD LCI Plan-Apochromat 25×/0.8 Imm Corr DIC M27 or a Plan-Apochromat 40×/1.4 Oil DIC M27 oil immersion or a 63×/1.3 Imm Corr DIC M27 LCI Plan-Neofluar (water) objective using standard settings.

### Whole-mount *in situ* RNA hybridization

RNA *in situ* hybridization in whole-mount embryos was performed as described previously [67] with minor alterations. Digoxigenin (DIG) labeled antisense RNA probes were generated with DIG RNA Labeling Kit (Sigma-Aldrich, Cat# 11175025910) from linearized *Np* and *tpr* cDNA. RNA hybridization in fixed embryos was performed at 70°C overnight. Anti-DIG-AP antibody (1:1000, Sigma-Aldrich, Cat# 11093274910) was used for detection of labelled RNA probes in NBT/BCIP Substrate Solution (Thermo Fisher Scientific, Cat# 34042). Images were acquired by bright field microscopy.

### Dextran permeability experiments

*mega*^*VE896*^/+ (control), *Np*^*P6*^ and *mega*^*VE896*^ embryos were collected at certain time points after egg laying (AEL), dechorionated and covered with Voltalef 10S oil for injection. Texas Red-labelled 10 kDa Dextran and Fluorescein-labelled 70 kDa dextran (Molecular Probes) were purified and injected into the haemocoel of embryos as described previously [33]. Embryos were analysed immediately after injection, and images were acquired by confocal microscopy.

### Cuticle preparations

Stage 17 wild-type and *Np* mutant embryos were dechorionated, mounted in Hoyer’s medium and incubated overnight at 65°C [70]. Images were acquired using dark field microscopy.

### Protein overexpression in cultured *Drosophila* cells

*D*. *melanogaster* S2R+ cells (DGRC) and Kc167 cells (DGRC) were kept in flasks containing Schneider's *Drosophila* medium (Thermo Fisher Scientific) supplemented with 1% Penicillin-Streptomycin (Thermo Fisher Scientific) and 10% FBS (Sigma-Aldrich) at 25°C. Confluent cells were detached, diluted 1:6, and transferred to a 10 cm diameter petri dish approximately 24 hours before transfection. Proteins were overexpressed using the Gal4/UAS system. Kc167 cells were transfected with either 1 μg *actin5C-Gal4* vector [71] and 1 μg *pUAST-Np-Strep*, 1 μg *actin5C-Gal4* vector and 1 μg *pUAST-Np*^*S990A*^-*Strep*, 1 μg *actin5C-Gal4* vector and 1μg *pUAST-matriptase-Strep*, or 1 μg *actin5C-Gal4* vector and 1 μg *pUAST-matriptase*^*S805A*^-*Strep* using Effectene transfection reagent (Qiagen) according to the supplier’s protocol. S2R+ cells were transfected with either 1 μg *actin5C-Gal4* vector and 1 μg *pUAST-pio-Flag*, 1 μg *actin5C-Gal4* vector and 1 μg *pUAST-pio*^*R196A*^-*Strep*, 1 μg *actin5C-Gal4* vector and 1 μg *pUAST-tpr-Flag* or 1 μg *actin5C-Gal4* vector and 1 μg *pUAST-tpr*^*ZL*^-*Flag* using Effectene transfection reagent (Qiagen) according to the supplier’s protocol. The medium of protease-expressing cells was removed 24 hours after transfection, cells were washed with PBS twice, and 6 ml Schneider's *Drosophila* medium supplemented with 1% Penicillin-Streptomycin was added.

### Protein purification and *in vitro* assays

Pio-Flag and Pio^R196A^-Flag-expressing cells were harvested 72 hours after transfection, washed twice with PBS, and lysed by pipetting up and down in 200 μl lysis buffer (10 mM Tris/Cl pH 7.5; 150 mM NaCl; 0.5% NP-40) for 30 min followed by subsequent centrifugation (15000 rpm; 10 min). Supernatants of Tpr-Flag and Tpr^ZL^-Flag-expressing cells were harvested 72 hours after transfection and centrifuged (900 rpm; 5min). Flag-tagged proteins were then purified using Anti-Flag Magnetic Agarose (Invitrogen, Cat# A36797) according to the supplier’s protocol.

Supernatants of cells that expressed Strep-tagged Np, Np^S990A^, matriptase, or matriptase^S805A^ were harvested 72 hours after transfection and centrifuged (900 rpm; 5 min). Proteases were then purified using MagStrep type3 XT Beads (Iba, Cat# 2-4090-002) according to supplier’s protocol.

*In vitro* assays were done in 15 μl reaction buffer (100mM Tris/Cl pH 8.5; 50 mM NaCl; 2mM CaCl) by using 1 μg of potential protease substrates (Pio-Flag, Pio^R196A^-Flag, Tpr-Flag or Tpr^ZL^-Flag) and adding 1 μg of either Np-Strep, Np^S990A^-Strep, matriptase-Strep, or matriptase^S805A^-Strep. Samples were incubated at 22°C over night and subsequently analysed by SDS-PAGE (denaturing and reducing conditions) followed by western blotting and immunostainings. For detection of proteins, mouse anti-Flag (1:10K; Sigma-Aldrich), mouse anti-Strep-HRP (1:10K; IBA Lifesciences) and sheep anti-human-matriptase (1:10K; R&D Systems) were used with goat anti-mouse-HRP (1:10K; Thermo Fisher Scientific) and mouse anti-sheep-HRP (1:10K; Jackson ImmunoResearch) as secondary antibodies.

### Electron microscopy

Stage 17 *Drosophila* embryos were dechorionated, transferred to a 150 μm specimen planchette (Engineering Office M. Wohlwend GmbH), and frozen with a Leica HBM 100 high-pressure freezer (Leica Microsystems). Vitrified samples were embedded with an Automatic Freeze Substitution Unit (AFS; Leica Microsystems) at -90°C in a solution containing anhydrous acetone, 0.1% tannic acid, and 0.5% glutaraldehyde for 72 hours and in anhydrous acetone, 2% OsO4, and 0.5% glutaraldehyde for additional 8 hours. Samples were then incubated at -20°C for 18 hours followed by warm-up to 4°C and subsequent washing with anhydrous acetone. Embedding in Agar 100 (Epon 812 equivalent) was performed at room temperature and polymerization at 60°C for 24 hours. Counterstaining of ultrathin sections was done with 1% uranylacetate in methanol. Images were taken in a Philips CM120 electron microscope (Philips Inc.) using a TemCam F416 CMOS camera (TVIPS).

### Quantification of tracheal gas filling and chitin organisation

For quantification of tracheal gas filling, embryos were aged until 23–24 hours AEL and analysed by bright field microscopy. For each genotype, 120 embryos were analysed for gas filling of the tracheal system. To quantify chitin organisation of the mature tracheal aECM, stage 17 embryos were stained with FITC-conjugated CBP and imaged by confocal microscopy. For each genotype, 40 embryos were analysed and defined as embryos with “wild-type like chitin organisation” if the chitin strands showed a parallel organisation perpendicular to the longitudinal dorsal trunk axis.

### Quantification and analysis of chitin strands in the embryonic tracheal aECM

Stage 17 embryos were stained with FITC-conjugated CBP and dorsal trunks were imaged using confocal microscopy. For each genotype, 10 embryos were analysed. For each embryo, two measurements of “chitin strands per 10 μm dorsal trunk length” and twenty “diameter of chitin strands” measurements were done using Fiji software [72]. Measurements were performed in dorsal trunk sections of abdominal segments 5–7. For “chitin strands per 10 μm dorsal trunk length” measurements, the chitin strands that were present in a 10 μm section of longitudinal dorsal trunk length were counted. Diameters of chitin strands were measured by plotting intensity profiles of the longitudinal dorsal trunk axis in images with focal planes of the chitin strands. Lengths of intensity peaks were then measured to determine the diameter of chitin strands.

## Supporting information

S1 Fig *Notopleural* mutant alleles and expression. (A-C) Generation of *Np* mutants by CRISPR/Cas9. We used CRISPR/Cas9 technology to generate frame shift mutations in the 5’ region of the *Np* open reading frame. (A) Physical map of genomic region 45A1 containing the *Np* gene. The single guide RNA recognition site (magenta letters) and PAM (green letters) are indicated. Translated DNA is indicated in black boxes. (B) Wild-type DNA sequences of the *Np* gene and the corresponding DNA deletions of *Np*^*P6*^ and *Np*^*C2*^ DNA are indicated. (C) Schemes of the predicted size of wild-type Np and the truncated Np^P6^ and Np^C2^ proteins. Yellow box indicates the putative transmembrane region, green box the catalytic domain of wild-type Np, and V the putative activation cleavage site. Red boxes indicate truncated protein sequences caused by the frame-shift mutations in the Np^P6^ and Np^C2^ proteins.
(D-I) Notopleural is expressed in ectodermally derived tissues. Whole-mount in situ hybridization of wild-type embryos with a digoxigenin-labelled *Np* antisense probe. (D) *Np* transcripts are first detectable during stage 11 in the tracheal placodes of the tracheal system (tr). (E) During stage 13, *Np* transcripts persist in the trachea and become visible in the foregut (fg), hindgut (hg), pharynx (ph), epidermis (ep), and posterior spiracles (ps). Tracheal expression is most prominent during stage 14 (F) and stage 15 (G, G’ different focal planes) and fades during stage 16 (H). Salivary gland (sg) expression is most prominent during stage 15 (G’).
(TIF)

S2 Fig Sequence alignment of the catalytic domains of Notopleural and matriptase. The catalytic domains of *Drosophila* Np and human matriptase reveal 41% sequence identity (red; asterisks) and additional 20% sequence similarity (colon).
(TIF)

S3 Fig Generation of GFP-tagged Notopleural by CRISPR/Cas9 technology. (A) Schematic overview of the *Np* genomic DNA region together with the donor vector containing two homology arms (red), the *white* gene, the attP site, and two loxP sites. The sgRNA recognition site is indicated in magenta letters.
(B) *Np* genomic region after CRISPR/Cas9-directed homology repair (top) and Cre recombinase-mediated white gene excision (bottom).
(C) Donor vector for φC31-integrase mediated integration (top) and generation of *white*^*+*^; *Np*::GFP allele (bottom).
(D) *Np*::GFP allele after Cre recombinase-mediated *white* gene excision.
(TIF)

S4 Fig Notopleural::GFP expression during embryogenesis. Confocal LSM images of whole-mount anti-GFP antibody stainings of *Np*::GFP embryos at stage 13 (A), 14 (B), 15 (C) and 16 (D). Abbreviations: sg, salivary gland; fg, foregut; hg, hindgut; tr, tracheal system; ps, posterior spiracles; ph, pharynx; ep, epidermis. Scale bars correspond to 50 μm.
(TIF)

S5 Fig The localisation of Kune-kune, DE-cadherin and Crumbs are not affected in *Notopleural* mutant embryos. Confocal LSM images of dorsal trunks of stage 16 wild-type (A-A”, C-C”) and *Np* mutant (B-B”, D-D”) embryos stained with anti-Kune-kune and anti-DE-cadherin antibodies (A-B”) or anti-Crumbs antibody and CBP (C-D”). Scale bars correspond to 10 μm.
(TIF)

S6 Fig Transepithelial barrier function of *Notopleural* and *megatrachea* mutant embryos. Confocal images of tracheal dorsal trunk branches of control (*mega/+*), *Np*, and *mega* mutant embryos at 18–19 h AEL (A, D, G, J, M, P), at 19–20 h AEL (B, E, H, K, N, Q) and at 20–21 h AEL (C, F, I, L, O, R) after 10 kDa Texas Red-dextran (A-C, E-I, K-R) and/or 70 kDa Fluorescein-dextran (B-F, H-I, J-R) injection. In control embryos (A-F), neither 10 kDa nor 70 kDa dextran diffuse into the tracheal lumen. In *mega* mutant embryos (M-R), 10 kDa and 70 kDa dextran diffuse into the tracheal lumen. In *Np* mutant embryos (G-L), neither 10 kDa nor 70 kDa dextran diffuse into the tracheal lumen of embryos at 18–19 h AEL (G, J). At 19–20 h AEL, 10 kDa dextran (H) but not 70 kDa dextran (K) diffuses into the tracheal lumen of *Np* mutant embryos. *Np* mutant embryos at 20–21 h AEL show no barrier function for 10 kDa and 70 kDa dextran (I, L). Scale bars correspond to 10 μm.
(TIF)

S7 Fig Sequence alignment of the catalytic domains of Notopleural and Lumens interrupted. The catalytic domains of Np and Lumens interrupted (Lint) reveal 44% sequence identity (red; asterisks) and additional 20% sequence similarity (colon).
(TIF)

S8 Fig Human matriptase rescues tracheal Dumpy degradation in *Np* mutant embryos. Confocal LSM images of dorsal trunks of stage 17 *dpy*::YFP, *Np* mutant embryos with tracheal expression (*btl*-Gal4) of UAS-*Np* (A-A”), UAS-matriptase (B-B”) or catalytically inactive UAS-*Np*^*S990A*^ (C-C”) stained with anti-GFP antibody and CBP. Luminal Dpy::YFP is degraded in embryos with tracheal expression of Np (A-A”) or matriptase (B-B”), but is not degraded in embryos with tracheal expression of Np^S990A^ (C-C”). Scale bars correspond to 10 μm.
(TIF)

S9 Fig Sequence alignment of the serine proteases Tracheal-prostasin and prostasin. The catalytic domains (grey highlight) of *Drosophila* Tpr and human prostasin reveal 35% sequence identity (red; asterisks) and additional 19% sequence similarity (colon).
(TIF)

S10 Fig *tracheal-prostasin* expression and generation of mutant alleles. (A-D) *tracheal-prostasin* is expressed in the embryonic tracheal system. Whole-mount *in situ* hybridization of wild-type embryos with a digoxigenin-labelled *tpr* antisense RNA probe. *tpr* transcripts are detectable in the embryonic tracheal system (tr) during stage 15 (A, B; ventral view A, dorsal view B), 16 (C) and 17 (D).
(E-G) Generation of *tpr* mutants by CRISPR/Cas9. We used CRISPR/Cas9 technology to generate frame-shift mutations in the 5’ region of the *tpr* open reading frame. (E) Physical map of genomic region 58A1 containing the *tpr* gene and the single guide RNA recognition site (magenta letters) and PAM (green letters). Translated DNA is indicated in black boxes. (F) Wild-type DNA sequences of the *tpr* gene and the corresponding DNA deletions of *tpr*^*D1*^ and *tpr*^*F1*^ DNA are indicated. (G) Schemes of the putative wild-type Tpr and the truncated Tpr^D1^ and Tpr^F1^ proteins. The predicted signal peptide (sp; blue), disulfide bridge (S-S), activation cleavage site (V), and catalytic protease domain (green) are indicated. Red boxes indicate truncated protein sequences caused by the frame-shift mutations in the Tpr^D1^ and Tpr^F1^ proteins.
(TIF)

S11 Fig Generation of RFP-tagged Tracheal-prostasin by CRISPR/Cas9 technology. (A) Schematic overview of the *tpr* genomic DNA region together with the donor vector containing two homology arms (red), *P3-DsRed* marker gene, the attP site, and two loxP sites.
(B) tpr genomic region after CRISPR/Cas9-directed homology repair (top) and Cre recombinase-mediated *P3-DsRed* gene excision (bottom).
(C) Donor vector for φC31 integrase-mediated *tpr*::RFP integration (top) and generation of *white*^*+*^; *tpr*::RFP allele (bottom). (D) *tpr*::RFP allele after Cre recombinase-mediated *white* gene excision.
(TIF)

S12 Fig Codon optimized sequences for human HAI-1 and HAI-2. ORFs of human HAI-1 and human HAI-2 that were codon optimized for *Drosophila* flanked by 5’ EcoRI (green) and 3’ KpnI (blue) endonuclease restriction sites are shown.
(TIF)

S1 Movie Degradation of luminal Dumpy and gas filling of the tracheal system. Time-lapse image sequence of a dorsal trunk of a stage 17 *dpy*::YFP/*dpy*::YFP embryo (embryo is at approx. 19.5 hours AEL at start of the movie) is shown. Luminal Dumpy::YFP (green) is degraded and the tracheal system subsequently fills with gas. Images were taken at 22°C by confocal microscopy. Scale bar corresponds to 10 μm.
(AVI)

S2 Movie Luminal Dumpy is not degraded in *Notopleural* mutant embryos. Time-lapse image sequence of a dorsal trunk of a stage 17 *Np*^*P6*^,*dpy*::YFP/ *Np*^*P6*^,*dpy*::YFP embryo (embryo is at approx. 19.5 hours AEL at start of the movie) is shown. Luminal Dumpy::YFP (green) is not degraded and the tracheal system does not fill with gas. Images were taken at 22°C by confocal microscopy. Scale bar corresponds to 10 μm.
(AVI)
