# Supplementary figures and images for: Conserved function of the matriptase-prostasin proteolytic cascade during epithelial morphogenesis
Source: PLoS Genet. 2019 Jan 2;15(1):e1007882. doi: 10.1371/journal.pgen.1007882 (PMC6331135; doi:10.1371/journal.pgen.1007882)

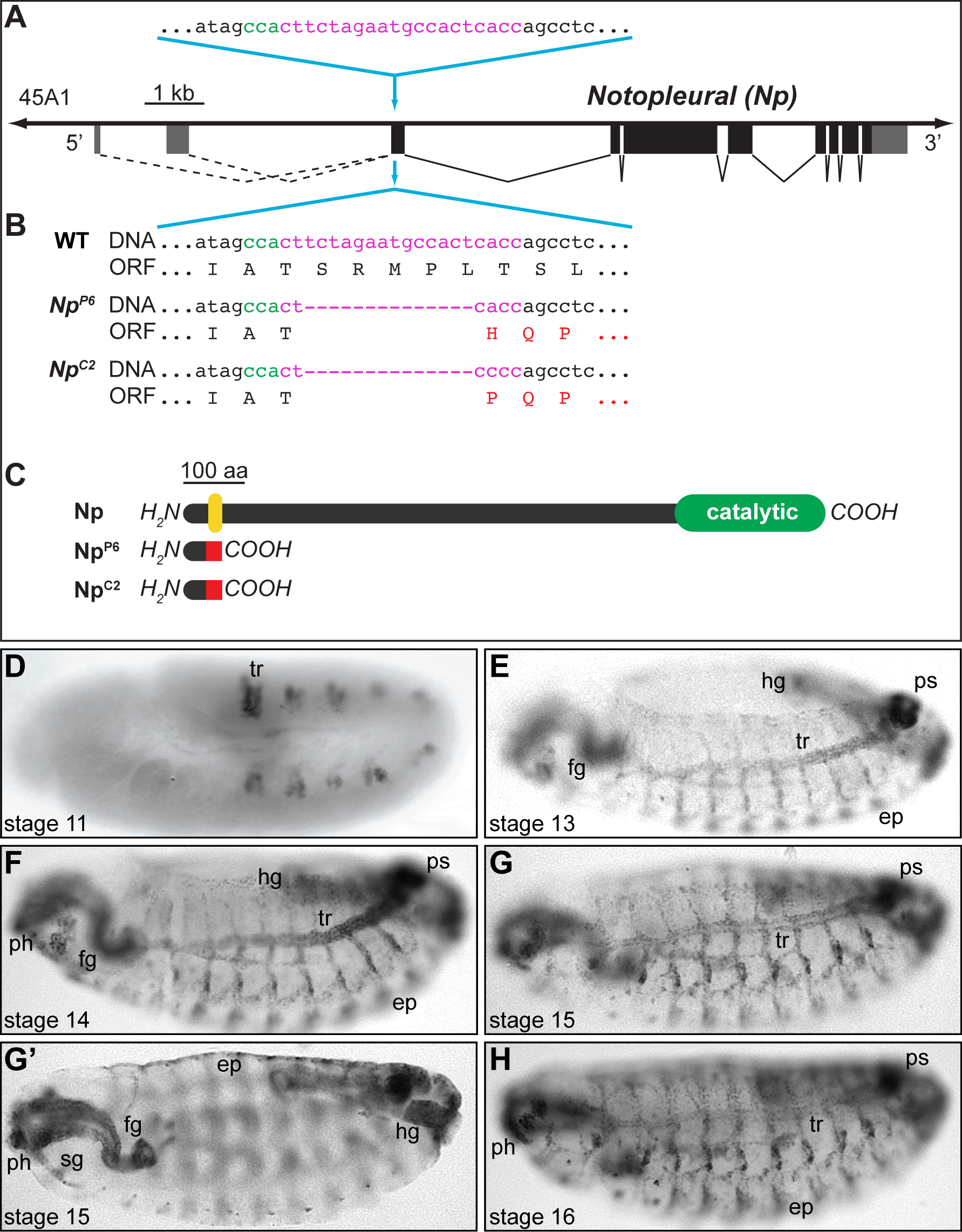

Supplement: S1 Fig — (A-C) Generation of Np mutants by CRISPR/Cas9. We used CRISPR/Cas9 technology to generate frame shift mutations in the 5’ region of the Np open reading frame. (A) Physical map of genomic region 45A1 containing the Np gene. The single guide RNA recognition site (magenta letters) and PAM (green letters) are indicated. Translated DNA is indicated in black boxes. (B) Wild-type DNA sequences of the Np gene and the corresponding DNA deletions of NpP6 and NpC2 DNA are indicated. (C) Schemes of the predicted size of wild-type Np and the truncated NpP6 and NpC2 proteins. Yellow box indicates the putative transmembrane region, green box the catalytic domain of wild-type Np, and V the putative activation cleavage site. Red boxes indicate truncated protein sequences caused by the frame-shift mutations in the NpP6 and NpC2 proteins. (D-I) Notopleural is expressed in ectodermally derived tissues. Whole-mount in situ hybridization of wild-type embryos with a digoxigenin-labelled Np antisense probe. (D) Np transcripts are first detectable during stage 11 in the tracheal placodes of the tracheal system (tr). (E) During stage 13, Np transcripts persist in the trachea and become visible in the foregut (fg), hindgut (hg), pharynx (ph), epidermis (ep), and posterior spiracles (ps). Tracheal expression is most prominent during stage 14 (F) and stage 15 (G, G’ different focal planes) and fades during stage 16 (H). Salivary gland (sg) expression is most prominent during stage 15 (G’). (TIF) [file pgen.1007882.s001.tif]

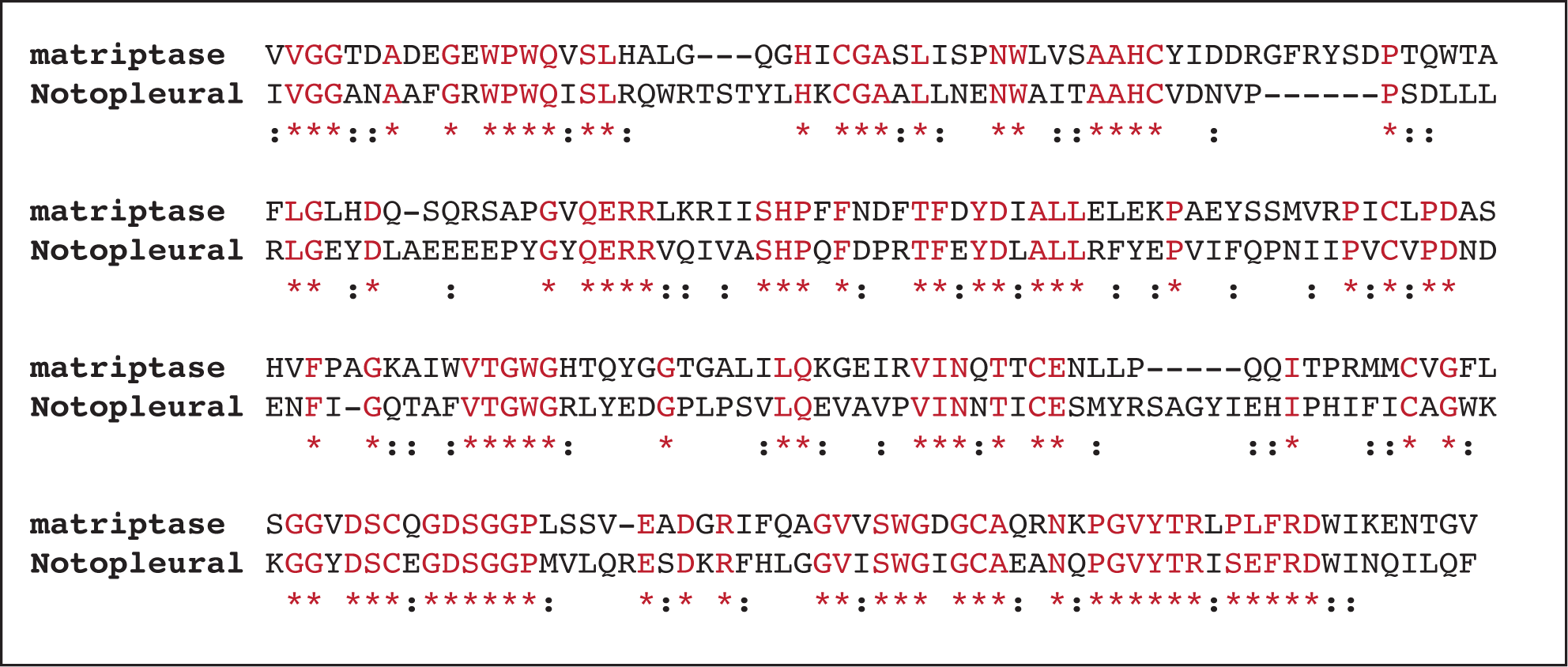

Supplement: S2 Fig — The catalytic domains of Drosophila Np and human matriptase reveal 41% sequence identity (red; asterisks) and additional 20% sequence similarity (colon). (TIF) [file pgen.1007882.s002.tif]

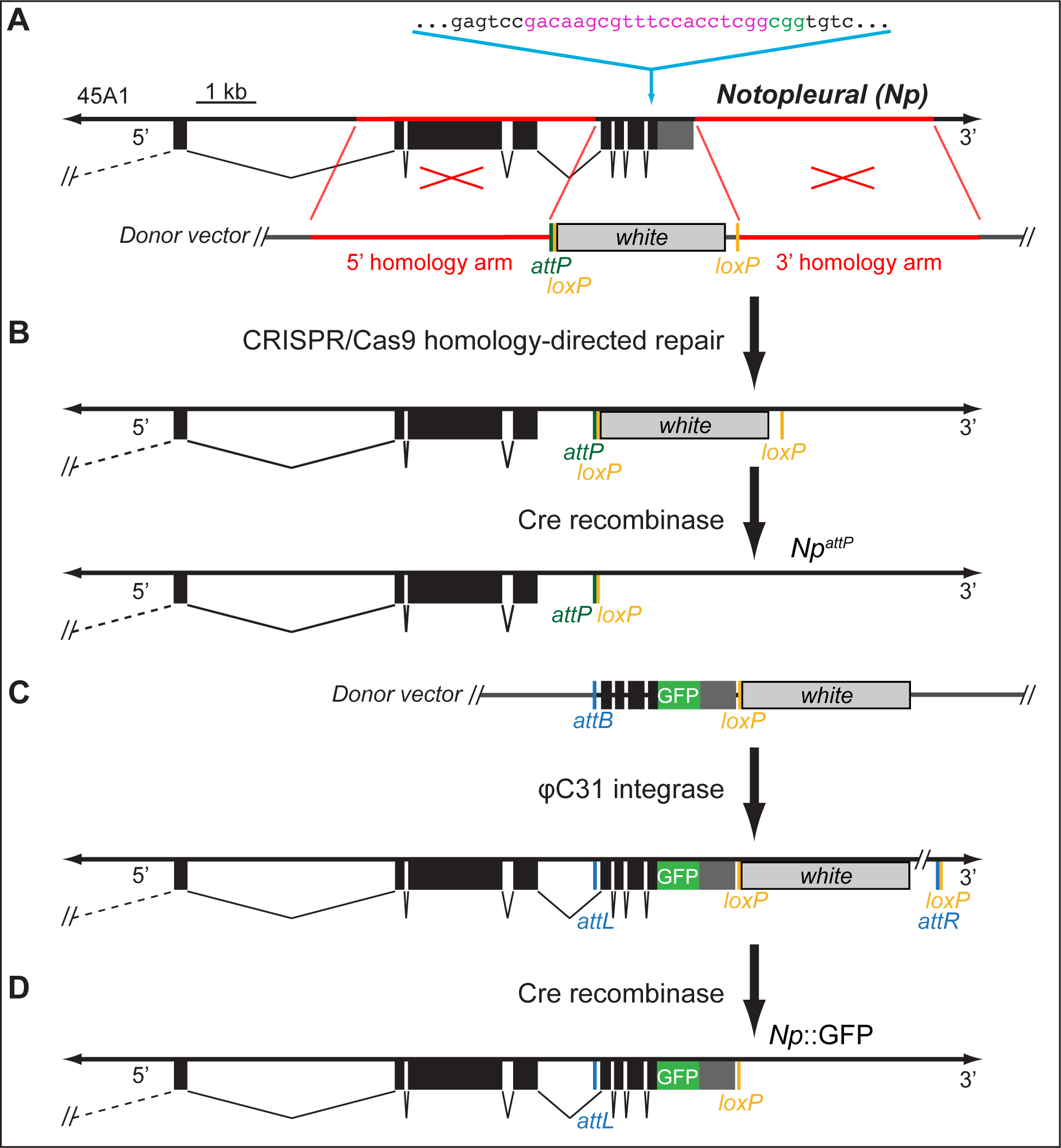

Supplement: S3 Fig — (A) Schematic overview of the Np genomic DNA region together with the donor vector containing two homology arms (red), the white gene, the attP site, and two loxP sites. The sgRNA recognition site is indicated in magenta letters. (B) Np genomic region after CRISPR/Cas9-directed homology repair (top) and Cre recombinase-mediated white gene excision (bottom). (C) Donor vector for φC31-integrase mediated integration (top) and generation of white+; Np::GFP allele (bottom). (D) Np::GFP allele after Cre recombinase-mediated white gene excision. (TIF) [file pgen.1007882.s003.tif]

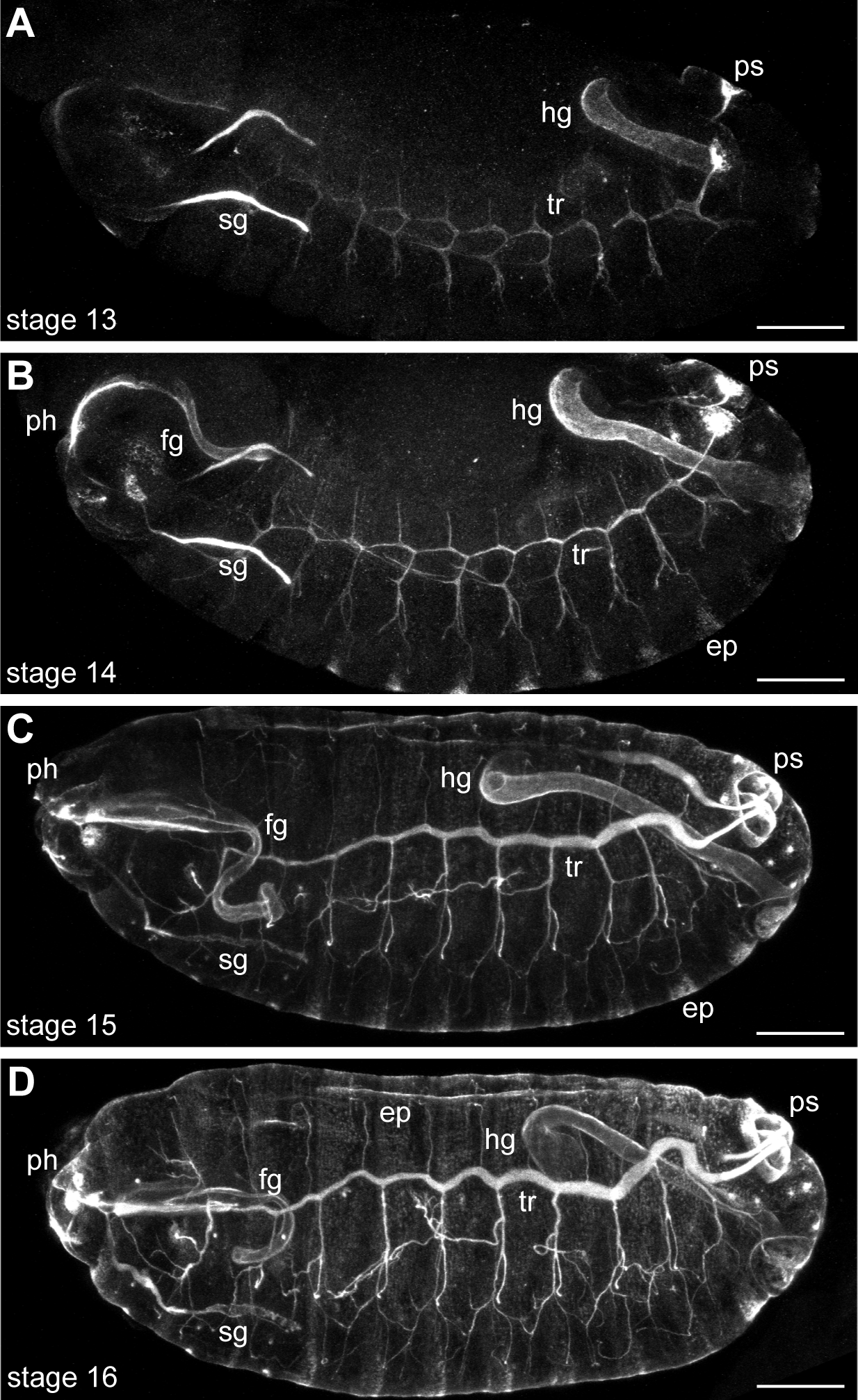

Supplement: S4 Fig — Confocal LSM images of whole-mount anti-GFP antibody stainings of Np::GFP embryos at stage 13 (A), 14 (B), 15 (C) and 16 (D). Abbreviations: sg, salivary gland; fg, foregut; hg, hindgut; tr, tracheal system; ps, posterior spiracles; ph, pharynx; ep, epidermis. Scale bars correspond to 50 μm. (TIF) [file pgen.1007882.s004.tif]

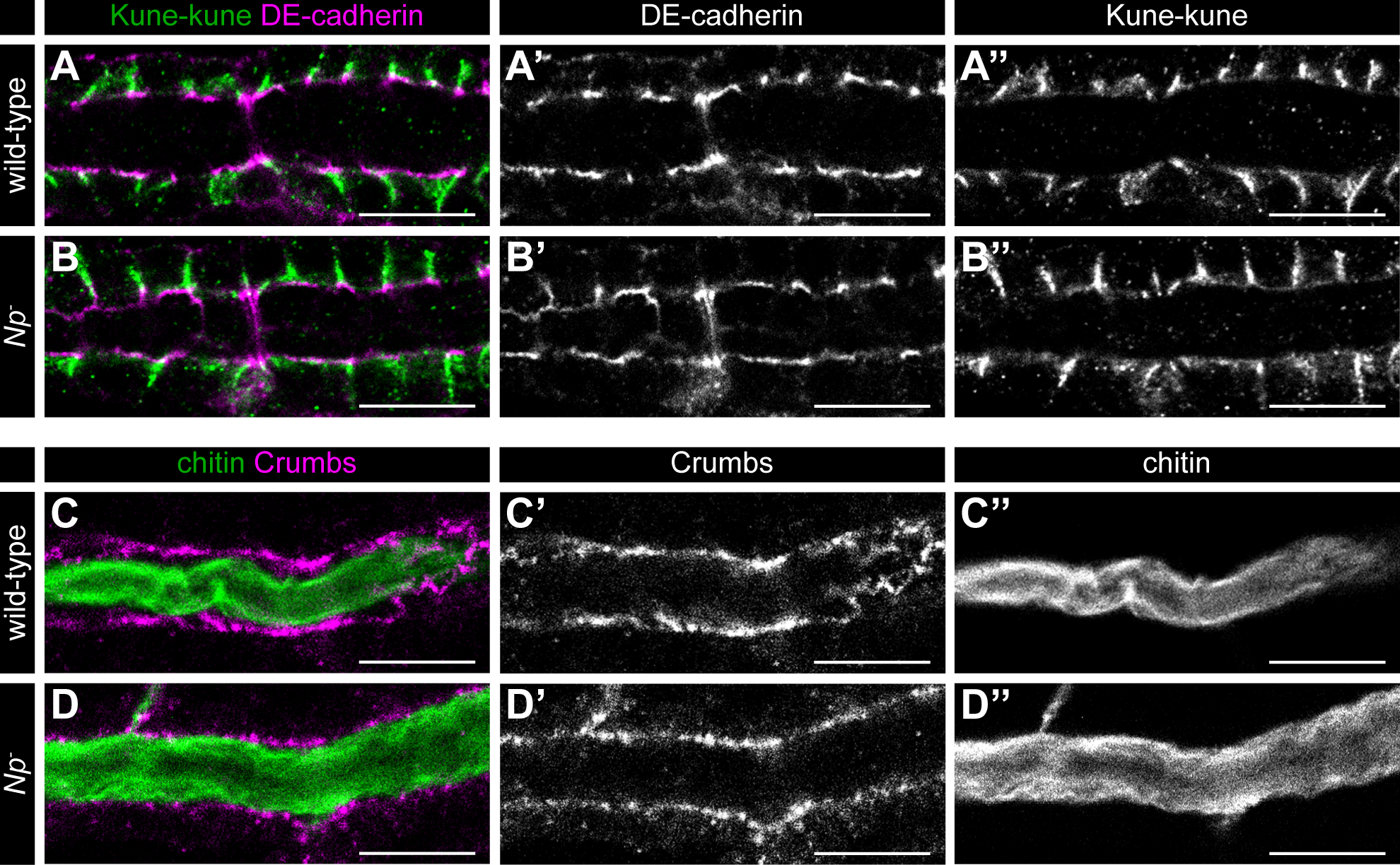

Supplement: S5 Fig — Confocal LSM images of dorsal trunks of stage 16 wild-type (A-A”, C-C”) and Np mutant (B-B”, D-D”) embryos stained with anti-Kune-kune and anti-DE-cadherin antibodies (A-B”) or anti-Crumbs antibody and CBP (C-D”). Scale bars correspond to 10 μm. (TIF) [file pgen.1007882.s005.tif]

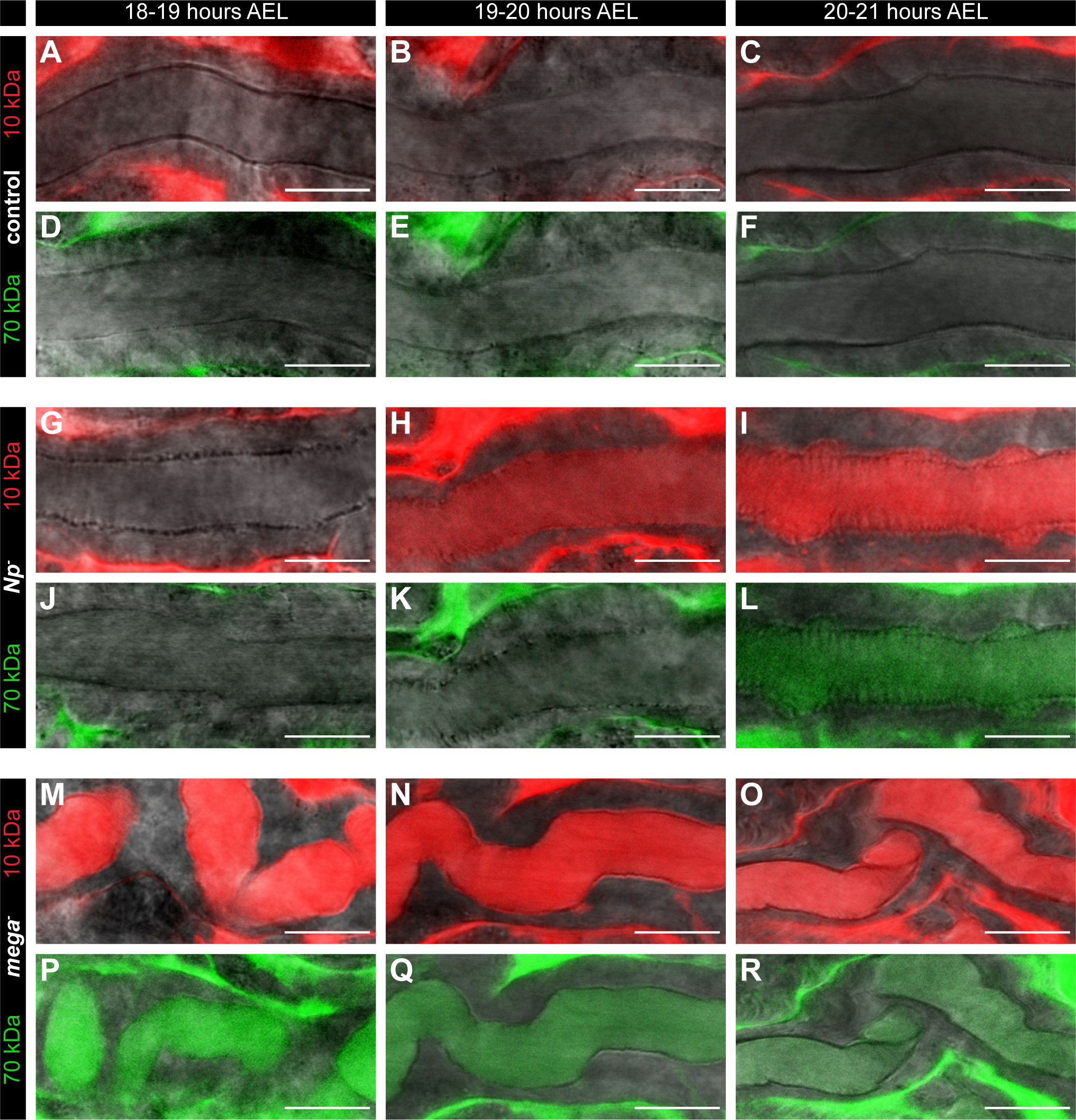

Supplement: S6 Fig — Confocal images of tracheal dorsal trunk branches of control (mega/+), Np, and mega mutant embryos at 18–19 h AEL (A, D, G, J, M, P), at 19–20 h AEL (B, E, H, K, N, Q) and at 20–21 h AEL (C, F, I, L, O, R) after 10 kDa Texas Red-dextran (A-C, E-I, K-R) and/or 70 kDa Fluorescein-dextran (B-F, H-I, J-R) injection. In control embryos (A-F), neither 10 kDa nor 70 kDa dextran diffuse into the tracheal lumen. In mega mutant embryos (M-R), 10 kDa and 70 kDa dextran diffuse into the tracheal lumen. In Np mutant embryos (G-L), neither 10 kDa nor 70 kDa dextran diffuse into the tracheal lumen of embryos at 18–19 h AEL (G, J). At 19–20 h AEL, 10 kDa dextran (H) but not 70 kDa dextran (K) diffuses into the tracheal lumen of Np mutant embryos. Np mutant embryos at 20–21 h AEL show no barrier function for 10 kDa and 70 kDa dextran (I, L). Scale bars correspond to 10 μm. (TIF) [file pgen.1007882.s006.tif]

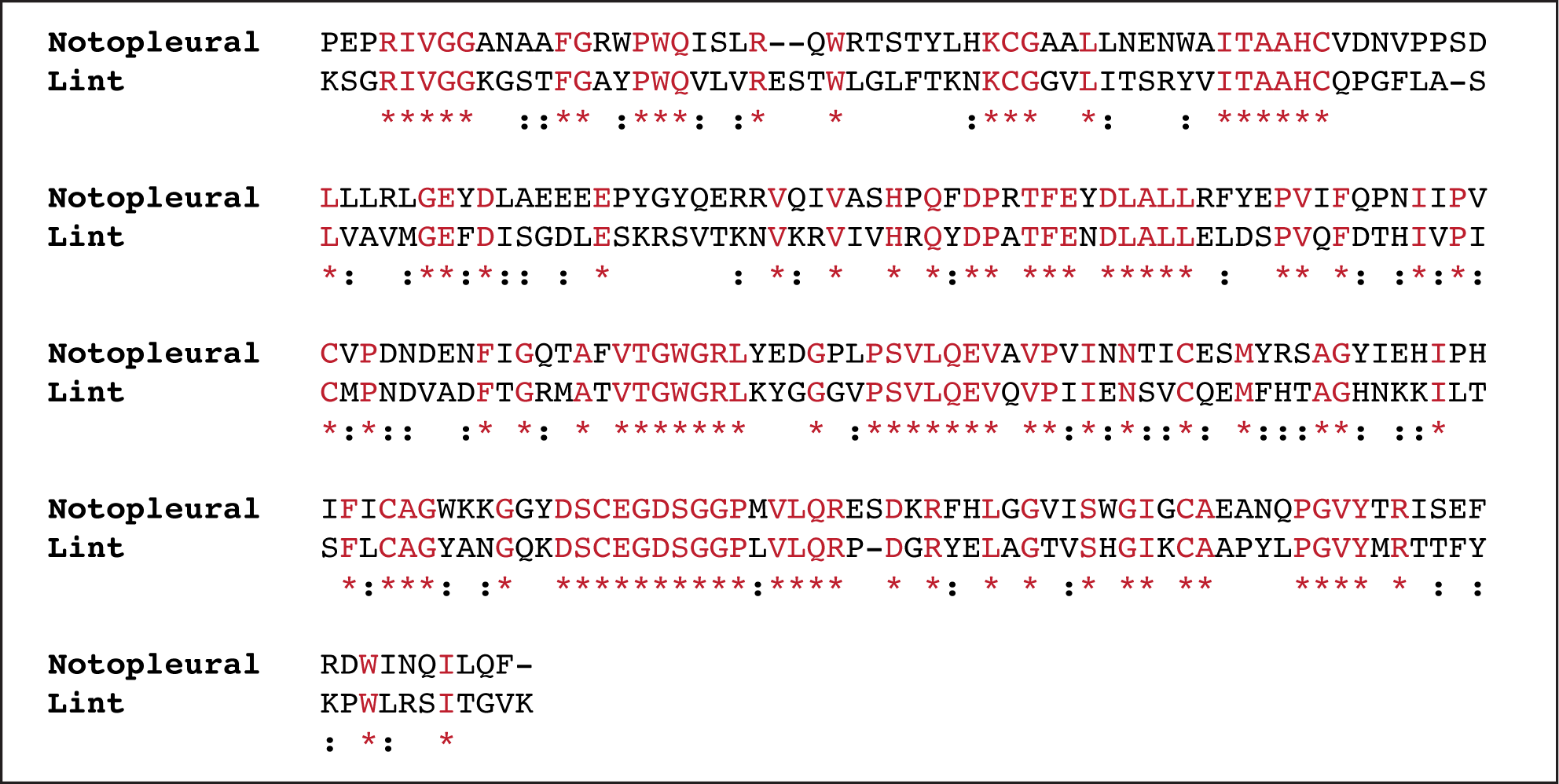

Supplement: S7 Fig — The catalytic domains of Np and Lumens interrupted (Lint) reveal 44% sequence identity (red; asterisks) and additional 20% sequence similarity (colon). (TIF) [file pgen.1007882.s007.tif]

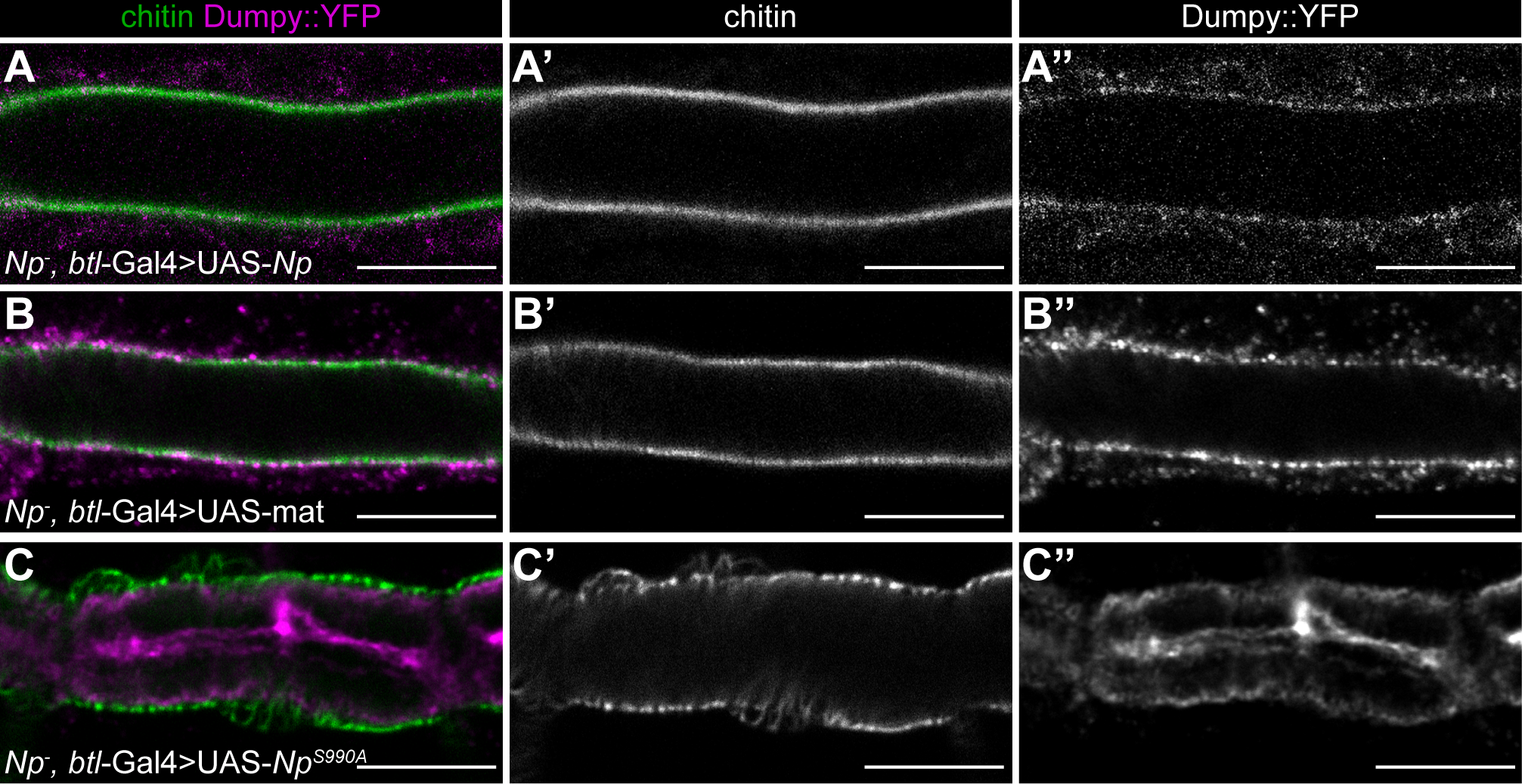

Supplement: S8 Fig — Confocal LSM images of dorsal trunks of stage 17 dpy::YFP, Np mutant embryos with tracheal expression (btl-Gal4) of UAS-Np (A-A”), UAS-matriptase (B-B”) or catalytically inactive UAS-NpS990A (C-C”) stained with anti-GFP antibody and CBP. Luminal Dpy::YFP is degraded in embryos with tracheal expression of Np (A-A”) or matriptase (B-B”), but is not degraded in embryos with tracheal expression of NpS990A (C-C”). Scale bars correspond to 10 μm. (TIF) [file pgen.1007882.s008.tif]

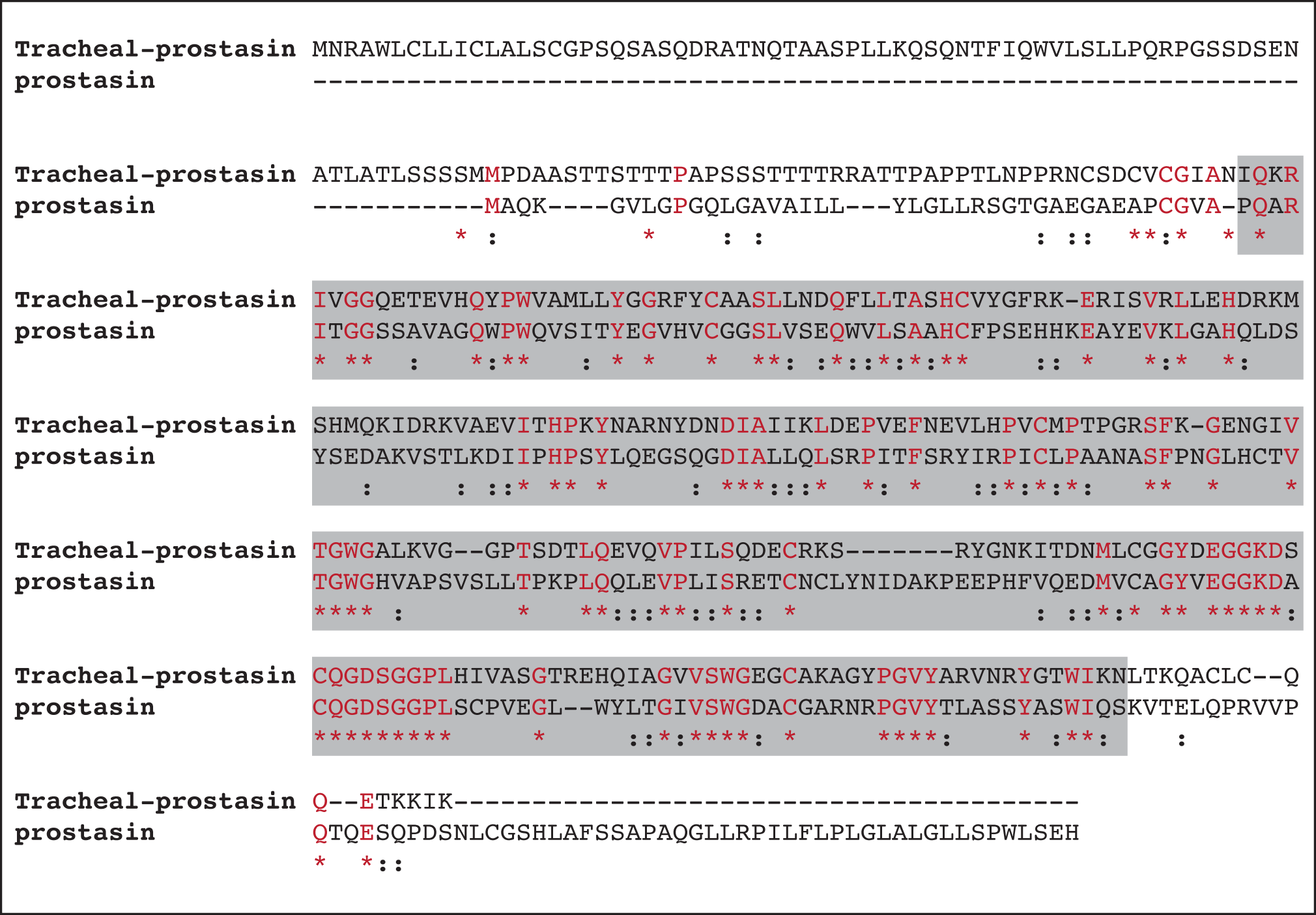

Supplement: S9 Fig — The catalytic domains (grey highlight) of Drosophila Tpr and human prostasin reveal 35% sequence identity (red; asterisks) and additional 19% sequence similarity (colon). (TIF) [file pgen.1007882.s009.tif]

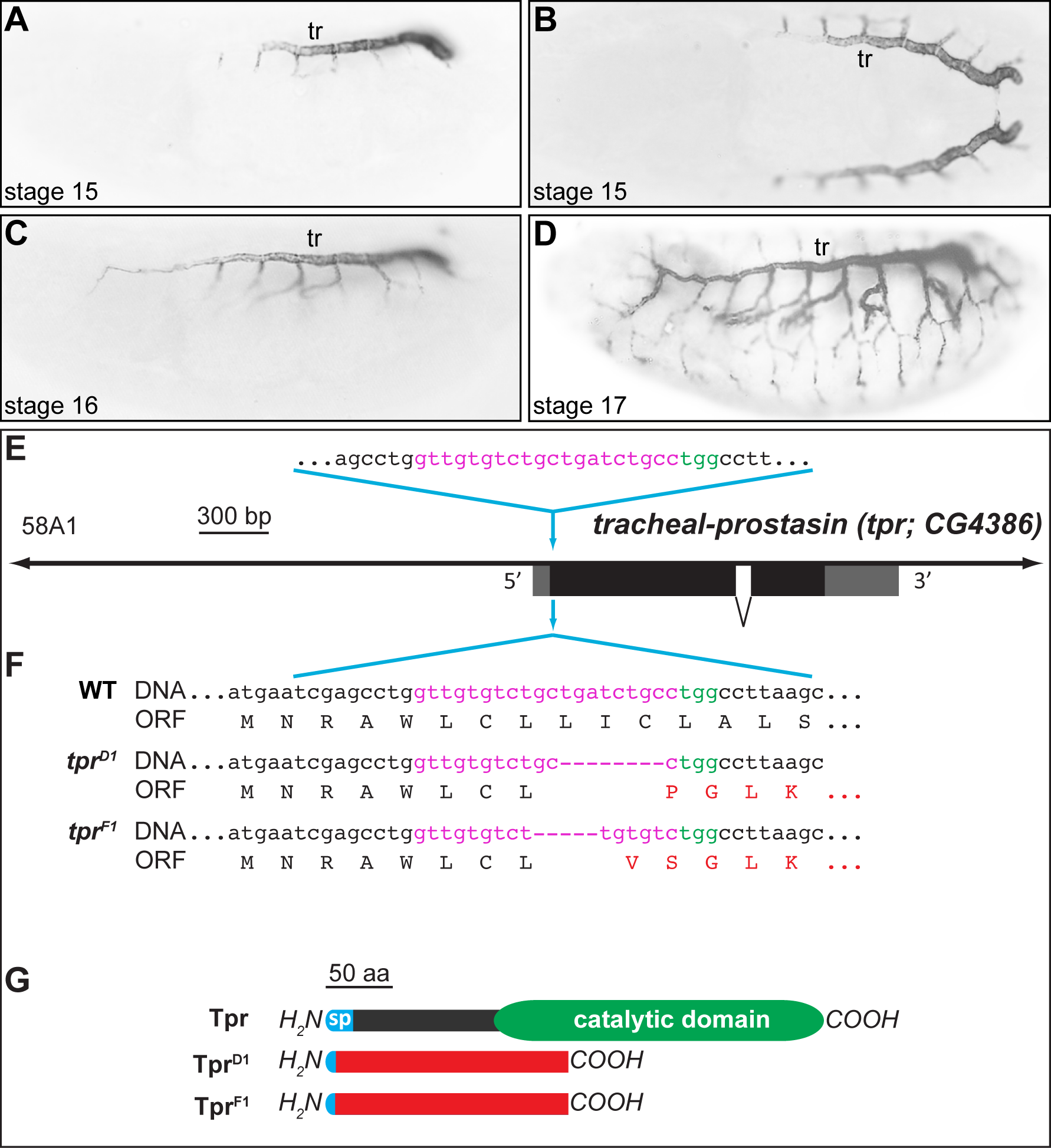

Supplement: S10 Fig — (A-D) tracheal-prostasin is expressed in the embryonic tracheal system. Whole-mount in situ hybridization of wild-type embryos with a digoxigenin-labelled tpr antisense RNA probe. tpr transcripts are detectable in the embryonic tracheal system (tr) during stage 15 (A, B; ventral view A, dorsal view B), 16 (C) and 17 (D). (E-G) Generation of tpr mutants by CRISPR/Cas9. We used CRISPR/Cas9 technology to generate frame-shift mutations in the 5’ region of the tpr open reading frame. (E) Physical map of genomic region 58A1 containing the tpr gene and the single guide RNA recognition site (magenta letters) and PAM (green letters). Translated DNA is indicated in black boxes. (F) Wild-type DNA sequences of the tpr gene and the corresponding DNA deletions of tprD1 and tprF1 DNA are indicated. (G) Schemes of the putative wild-type Tpr and the truncated TprD1 and TprF1 proteins. The predicted signal peptide (sp; blue), disulfide bridge (S-S), activation cleavage site (V), and catalytic protease domain (green) are indicated. Red boxes indicate truncated protein sequences caused by the frame-shift mutations in the TprD1 and TprF1 proteins. (TIF) [file pgen.1007882.s010.tif]

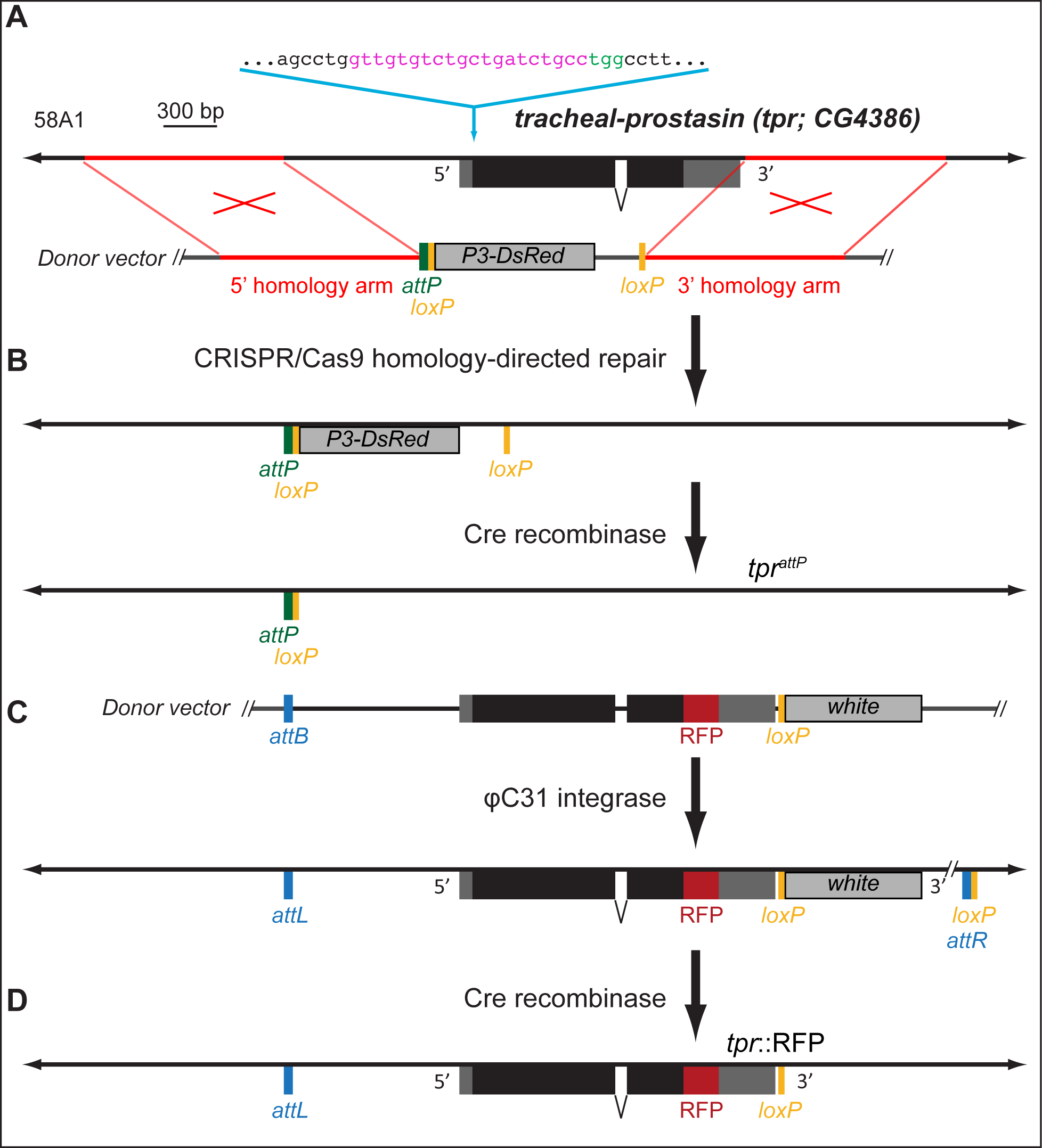

Supplement: S11 Fig — (A) Schematic overview of the tpr genomic DNA region together with the donor vector containing two homology arms (red), P3-DsRed marker gene, the attP site, and two loxP sites. (B) tpr genomic region after CRISPR/Cas9-directed homology repair (top) and Cre recombinase-mediated P3-DsRed gene excision (bottom). (C) Donor vector for φC31 integrase-mediated tpr::RFP integration (top) and generation of white+; tpr::RFP allele (bottom). (D) tpr::RFP allele after Cre recombinase-mediated white gene excision. (TIF) [file pgen.1007882.s011.tif]

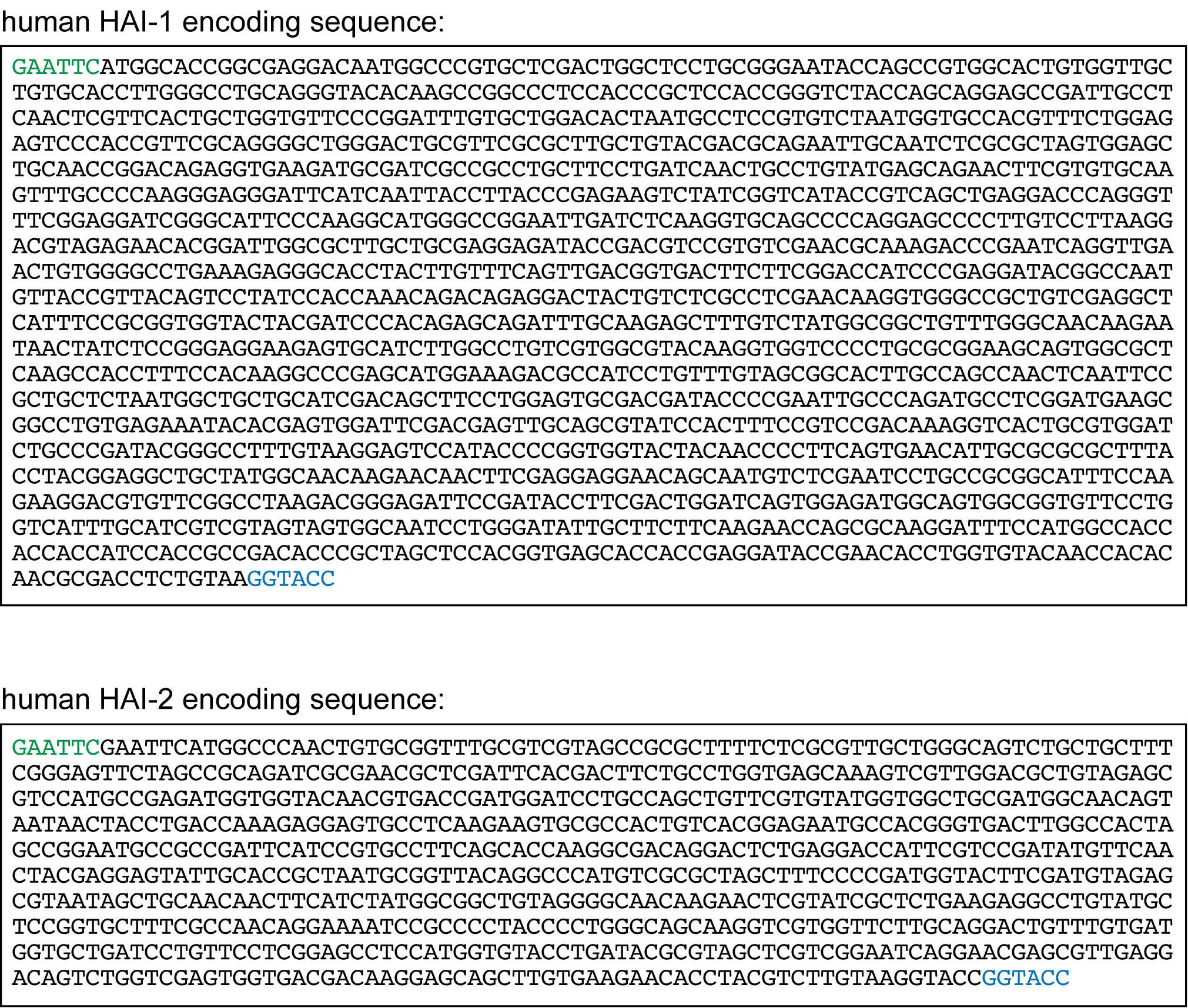

Supplement: S12 Fig — ORFs of human HAI-1 and human HAI-2 that were codon optimized for Drosophila flanked by 5’ EcoRI (green) and 3’ KpnI (blue) endonuclease restriction sites are shown. (TIF) [file pgen.1007882.s012.tif]
